# Cloning and Characterization of *Trypanosoma congolense* and *T. vivax* Nucleoside Transporters Reveal the Potential of P1-Type Carriers for the Discovery of Broad-Spectrum Nucleoside-Based Therapeutics against Animal African Trypanosomiasis

**DOI:** 10.3390/ijms24043144

**Published:** 2023-02-05

**Authors:** Marzuq A. Ungogo, Mustafa M. Aldfer, Manal J. Natto, Hainan Zhuang, Robyn Chisholm, Katy Walsh, MarieClaire McGee, Kayhan Ilbeigi, Jamal Ibrahim Asseri, Richard J. S. Burchmore, Guy Caljon, Serge Van Calenbergh, Harry P. De Koning

**Affiliations:** 1 School of Infection and Immunity, College of Medical, Veterinary and Life Sciences, University of Glasgow, Glasgow G12 8TA, UK; 2 Department of Veterinary Pharmacology and Toxicology, Ahmadu Bello University, Zaria 810107, Kaduna State, Nigeria; 3 Roslin Institute, Royal (Dick) School of Veterinary Studies, University of Edinburgh, Midlothian EH25 9RG, UK; 4 Laboratory of Microbiology, Parasitology and Hygiene (LMPH), University of Antwerp, B-2610 Wilrijk, Belgium; 5 Laboratory for Medicinal Chemistry (Campus Heymans), Ghent University, B-9000 Gent, Belgium

**Keywords:** *Trypanosoma vivax*, *Trypanosoma congolense*, nucleoside antimetabolite, adenosine transporter, nucleoside transporter, animal trypanosomiasis, nagana, tubercidin analog

## Abstract

African Animal Trypanosomiasis (AAT), caused predominantly by *Trypanosoma brucei brucei*, *T. vivax* and *T. congolense*, is a fatal livestock disease throughout Sub-Saharan Africa. Treatment options are very limited and threatened by resistance. Tubercidin (7-deazaadenosine) analogs have shown activity against individual parasites but viable chemotherapy must be active against all three species. Divergence in sensitivity to nucleoside antimetabolites could be caused by differences in nucleoside transporters. Having previously characterized the *T. brucei* nucleoside carriers, we here report the functional expression and characterization of the main adenosine transporters of *T. vivax* (TvxNT3) and *T. congolense* (TcoAT1/NT10), in a *Leishmania mexicana* cell line (‘SUPKO’) lacking adenosine uptake. Both carriers were similar to the *T. brucei* P1-type transporters and bind adenosine mostly through interactions with N3, N7 and 3′-OH. Expression of TvxNT3 and TcoAT1 sensitized SUPKO cells to various 7-substituted tubercidins and other nucleoside analogs although tubercidin itself is a poor substrate for P1-type transporters. Individual nucleoside EC_50_s were similar for *T. b. brucei*, *T. congolense*, *T. evansi* and *T. equiperdum* but correlated less well with *T. vivax*. However, multiple nucleosides including 7-halogentubercidines displayed pEC50>7 for all species and, based on transporter and anti-parasite SAR analyses, we conclude that nucleoside chemotherapy for AAT is viable.

## 1. Introduction

African Animal Trypanosomiasis (AAT) is caused by multiple *Trypanosoma* species including *T. brucei brucei*, *T. b. rhodesiense*, *T. congolense*, *T. evansi*, *T. equiperdum* and *T. vivax*, and has remained one of the most important livestock diseases in sub-Saharan Africa, where it causes direct and indirect losses amounting to USD 4.75 billion annually [1]. Although the related human disease HAT has been targeted for elimination by the WHO by 2030 [2], it appears that AAT will continue to be around at least for decades. Despite some promising progress for *T. vivax*, implementation of a vaccine is at best years away, even for any single *Trypanosoma* species [3,4,5]. Control by chemotherapy is hampered by the paucity of drugs, and the widespread resistance to them [6,7]. This would leave control of the tsetse fly vector as the main option for AAT control, but this is difficult in the vast rural areas where it would be required and is further challenged by the spread of mechanically transmitted *T. vivax* and *T. evansi*, and the sexually transmitted *T. equiperdum*, inside and outside Africa [8,9,10]. Thus, tsetse control, while important, will not be enough. This, coupled with the problem of drug resistance [11,12], emphasizes the need for more effort in the study of veterinary trypanosomes [13].

In the last 60 years, only one new veterinary trypanocide has been introduced to the field, melarsomine (Cymelarsan, MelCy), which was developed in the late 1980s [14]; the other veterinary trypanocides in use are all 60 years old or more [15]. Therefore, the need for safer and more effective trypanocides cannot be overemphasized, especially considering the slim chances of vaccine development [5]. Furthermore, different isolates of veterinary trypanosomes have shown great variability in their drug sensitivity profile, which often leads to treatment failure and devastating impact on agricultural and rural economy of developing countries [11,16]. However, with the genetic differences between the three species causing AAT becoming clearer, it appears that differences in drug sensitivity can result from differences in their capacity to internalize the drugs [17,18] and loss of the unique transporters involved often contributes to drug resistance [19,20,21,22]. It is thus clear that in AAT drug design, one should consider exploiting the specific transporters for guiding drugs to their intracellular targets. This is important given that drug transport directly through the phospholipid membrane, i.e., by simple diffusion, is now believed to be negligible [23].

Purine salvage is an essential feature in protozoan parasites, which lack the capacity for the de novo synthesis of this essential nutrient [24,25]. Therefore, these parasites have developed highly specialized transporters in their membrane for the internalization of the hydrophilic nucleosides from the host environment [26]. To date, all protozoan purine and pyrimidine transporter genes identified have been of the Equilibrative Nucleoside Transporter (ENT) family [25,26,27,28,29,30], although there is a quite strong likelihood that some protozoan purine and/or pyrimidine carriers are expressed by other gene families [31,32]. Where this has been investigated, it was found that the trypanosomatid nucleoside transporters are concentrative proton symporters that exclude uric acid; acidification of the cytosol is prevented by H^+^-ATPases in the plasma membrane [33,34,35]. This feature distinguishes protozoan from mammalian ENT transporters, which allow only facilitated diffusion [36,37]. The coupling to the protonmotive force provides the energy for active transport, concentrating the substrate in the cell and making it possible to selectively target the transporters for the highly effective accumulation of toxic nucleoside drugs in trypanosomatids [26,38].

*Brucei*-group trypanosomes express two types of nucleoside transporters: multiple adenosine/inosine/guanosine carriers (P1-type) and the adenosine/adenine transporter (P2), encoded by a single gene [39,40,41,42]. The P2 transporter interacts with its substrates through the unprotonated N1, C6-NH_2_ and N9 of the purine ring [43,44,45], making it highly effective for the uptake of the toxic nucleosides tubercidin, cordycepin [46,47] and the 3′-deoxy-7-deazaadenosine analogs that combine the features of both nucleoside antibiotics [38,48]. Moreover, the transport of currently used drugs including diamidines, pentamidine and melaminophenyl arsenicals in *brucei* group trypanosomes depends on the P2 transporter [20,49,50,51]. However, the P1 transporter can also be critical for the uptake of other toxic nucleosides through interaction with the N3, N7, 3′-OH and 5′-OH functional groups [26,39,40,44]. Therefore, while *T. congolense* and *T. vivax* lack orthologues of the P2 transporter [52], the presence of a P1-type transporter in all three species causing AAT would present a unique chemotherapeutic potential.

Phylogenetic analysis has revealed the presence of a single nucleoside transporter gene in *T. congolense* (named *TcoAT1* or *TcoNT10*) and identified four putative nucleoside transporter genes in *T. vivax* genome [52,53]. TcoAT1 was earlier hypothesized to be a P2-type transporter and a determinant of drug resistance in *T. congolense,* based on the identification of mutations in the gene encoding the transporter, in diminazene-resistant field isolates [54]. Already diagnostic tests for the detection of a single point mutation in *TcoAT1* have been developed and improved [54,55], and applied either alone or in combination with other methods [56,57,58]. However, the expression of the *TcoAT1* gene in B48, a multidrug-resistant *T. brucei* strain lacking *TbAT1* [59], did not result in any significant increase in uptake or sensitivity to diminazene, pentamidine or melarsomine [52]. More recently, laboratory-generated diminazene resistant strains of *T. congolense* did not show a reduced uptake of diminazene compared to the diminazene-sensitive control and were devoid of mutations in the *TcoAT1* gene [60]. The functional characterization thus shows that TcoAT1 is a P1-type rather than a P2-type nucleoside transporter. Despite the interest in TcoAT1, the transporter’s substrate affinity and chemotherapeutic potential are yet to be fully characterized.

Four putative *T. vivax* ENT genes are suggested to encode for P1-type transporters due to their phylogenetic proximity to *T. congolense*, *T. cruzi* and *T. brucei* P1 transporters [52,53]. However, these *T. vivax* putative ENT’s have not been studied to date. Other pathogenic trypanosomatids such as *T. cruzi* and *Leishmania* spp. also express several P1-type nucleoside transporters [27,61]. *Leishmania* spp. nucleoside transporters (NT1 and NT2) are evolutionary conserved in *L. donovani, L. major* and *L. mexicana*; *Leishmania* NT1 is an adenosine and pyrimidine nucleoside transporter that is encoded by two closely related genes (NT1.1 and NT1.2), while NT2 transports inosine and guanosine [61,62,63]. Thus, broad-spectrum nucleoside transporters are common among kinetoplastid parasites while the P2 adenosine/adenine transporter is restricted to *brucei*-group trypanosomes.

We recently reported a sequential knock-out of the *LmexNT1.1*, *NT1.2* and *NT2* genes using CRISPR/Cas9 technology [64] in promastigotes, resulting in a mutant model with null background purine and pyrimidine nucleoside uptake named SUPKO, which was validated as a surrogate system that allows the functional transgenic expression and characterization of protozoan nucleoside transporters [29]. Here, we use this system to express *TcoAT1* and the putative *T. vivax* ENTs in order to establish their substrate preferences. In parallel, we studied the transport of adenosine in bloodstream forms of *T. congolense* to establish whether this activity can be ascribed to TcoAT1. The ultimate aim of this effort is to investigate whether there are nucleoside transporters in all of the main *Trypanosoma* spp. that cause AAT (*T. brucei*, *T. congolense*, *T. vivax*) that display sufficiently similar substrate preferences so that the same (class of) nucleoside analogs will be efficiently taken up by all three species, with the hope of finding nucleoside antimetabolites that can effectively treat AAT caused by all three species. That could lead to a treatment of AAT regardless of the infecting species. Nucleoside analogs have recently emerged as potent agents against protozoan parasites including *T. brucei*, *T. cruzi*, *T. congolense* and *Trichomonas vaginalis* [39,48,65,66,67,68], but the first efforts to identify analogs that are active against all three AAT pathogens had only limited success [69].

## 2. Results

### 2.1. Nucleoside Transport Activity in T. congolense

The genome of *T. congolense* contains 17 ENT-family transporter genes but only one aligns phylogenetically with the well-characterized nucleoside transporters of other kinetoplastid species, the others with the *T. brucei* NT8/NBT1 nucleobase transporters [70,71] and the *T. cruzi* NB1 hypoxanthine/guanine transporter [27,53]. The 16 putative nucleobase transporter genes divide into two distinct clades of 8 genes each (Supplemental Figure S1). Clade 1 consists of 8 closely related genes, 7 of which encode a protein of 339 amino acids, the last of 352 amino acids. Importantly, each are predicted to have just six transmembrane domains (TMDs), which makes it very unlikely that they are able to function as ENT transporters, which typically have 10 or 11 TMDs [37]. The second clade contains 7 genes encoding full-length transporters of 441–447 amino acids and 10 or 11 TMDs, as well as 1 apparently truncated gene encoding a protein of 320 amino acids and just 7 TMDs (Supplemental Table S1). A multiple alignment of the *T. congolense* nucleobase transporters shows that the Clade 1 genes lack the canonical C-terminal sequence of ~100 amino acids (Supplemental Figure S2). This analysis shows that the *T. congolense* reference genome contains 7 putative nucleobase transporters, 1 nucleoside transporter and 9 apparently truncated copies that are unlikely to contribute to pyrimidine or purine uptake.

**Figure 1:**
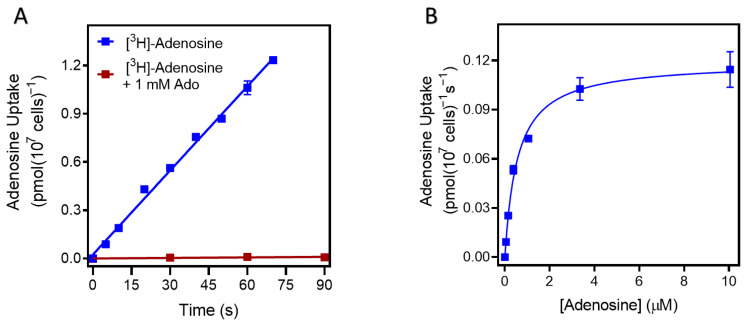
The uptake of 0.05 µM [^3^H]-adenosine in *T. congolense* IL3000. (**A**) Uptake over time, and (**B**) uptake at a fixed time point of 30 s, in the presence of unlabeled adenosine for the K_m_ determination, using 0–1 mM adenosine in bloodstream forms of *T. congolense*. Graphs show one representative experiment out of two (panel **A**) or three (panel **B**) independent repeats, each carried out in triplicate. Error bars represent standard error of the mean (SEM).

The sole *T. congolense* nucleoside transporter, TcoAT1/TcoNT10 (TcIL3000_9_2500 or TcIL3000.A.H_000665800; TriTrypDB), was shown to increase the transport of adenosine and inosine when expressed in a *T. brucei* surrogate [52]. However, nucleoside transport in cultured or ex vivo *T. congolense* cells has not yet been reported. We determined the rate of adenosine uptake in cultured *T. congolense* IL3000 as 0.014 ± 0.0037 pmol(10^7^ cells)^−1^s^−1^ (n = 2). Uptake of 50 nM [^3^H]-adenosine was linear (r^2^ = 0.996) over 70 s and was saturable, as transport in the presence of 1 mM unlabeled adenosine was not significantly different from zero (*p* = 0.18, F-test) (Figure 1A). Thus, a 30 s incubation time was selected for further inhibition studies in *T. congolense*, as being well inside the linear phase of uptake. This allowed for the transporter’s K_m_ for adenosine to be determined as 0.48 ± 0.03 µM and the V_max_ as 0.11 ± 0.02 pmol(10^7^ cells)^−1^s^−1^ (n = 3) (Figure 1B). In each experiment, the Hill slope was very close to −1 consistent with a single transporter being responsible for the adenosine uptake observed; 1 mM adenosine saturated the transporter completely (Figure 1A,B). In addition, inosine and guanosine inhibited adenosine uptake with K_i_ values of 0.27 ± 0.02 µM and 1.06 ± 0.07 µM, respectively. However, the pyrimidines cytidine, thymidine and uridine showed poor inhibition of adenosine uptake with Ki values >90-fold higher than those for the purines (Table 1).

The previously reported K_i_ values for P1-mediated uptake in *T. b. brucei* bloodstream forms [44] were also included in this table. It can be seen that the affinity for both purine and pyrimidine nucleosides was very similar for both transport systems. Indeed, as in *T. b. brucei* P1, N7 of the purine ring and the 3′-hydroxy group are shown to be important for adenosine binding by the *T. congolense* adenosine transporter as shown by the reduced affinity for tubercidin (7-deazaadenosine) and cordycepin (3′-deoxyadenosine), respectively. This profile is in sharp contrast to the substrate preferences of the *T. b. brucei* P2 transporter, which has high affinity for adenosine, cordycepin, tubercidin and adenine but not for inosine and guanosine [44,47], and we conclude that nucleoside salvage in *T. congolense* is through a P1-type transporter.

### 2.2. Expression of T. congolense and T. vivax Nucleoside Transporters in a L. mexicana NT-Null Cell Line

#### 2.2.1. Nucleoside Transporters of *T. vivax*

In *T. congolense,* only TcoAT1 is identified as a nucleoside transporter. On the other hand, four of the putative ENT-family genes in *T. vivax* showed closest proximity to *T. brucei* and *T. congolense* P1-type nucleoside transporter clade while three other ENT-family genes and two shorter fragments aligned best with the nucleobase transporter gene cluster (Table 2) [52,53]. The ENT-family genes of *T. vivax* were given numerical names TvxNT1—TvNT7, with the first four aligning with nucleoside transporters and the other three as nucleobase transporters. TvxNT4 lacked the C-terminal sequence of ~100 amino acids, much like some of the *T. congolense* nucleobase transporters discussed above, and it remains to be seen whether any one of them is functional. TvxNT5 has an intact C-terminal sequence but misses the first 51 amino acids including TMD1. It seems possible that some of these truncations may be due to inaccuracies in genome assembly.

**Figure 2:**
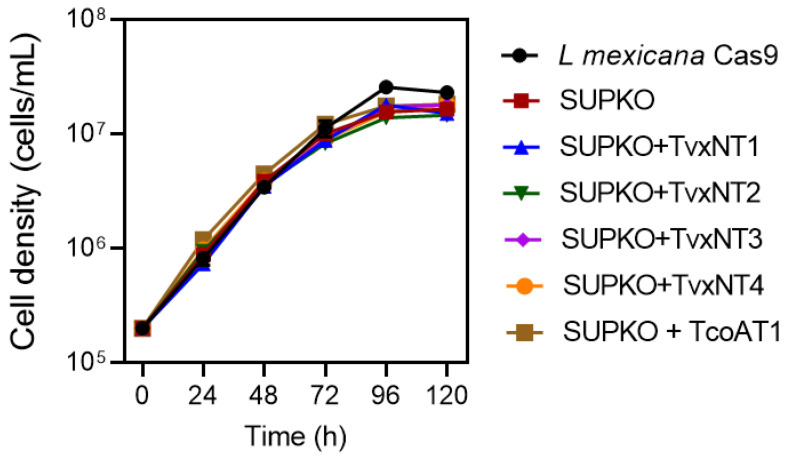
Growth curve of *L. mexicana* Cas9, SUPKO, and SUPKO clones expressing *T. congolense* and *T. vivax* nucleoside transporters. Cells were seeded at density of 2 × 10^5^ cells/mL in HOMEM medium and counted manually in a Neubauer chamber viewed under microscope every 24 h. Graph was plotted using the average count of three independent determinations (Mean ± SEM; n = 3). When error bars are not shown they fall within the symbol.

We have earlier reported the successful characterization of trypanosomatid nucleoside transporters by cloning and expression in *T. brucei* TbAT1-KO and TbNBT1-KO surrogates [31,52]. However, both surrogates exhibited background nucleoside and nucleotide uptake, thus limiting the extent to which the exogenous transporter can be characterized. Here we used a *L. mexicana* promastigote line with null nucleoside (NT1.1, NT1.2 and NT2-KO; ‘SUPKO’) or null nucleobase uptake (NT3-KO) as surrogates [29]. *TcoAT1* and each of the four *T. vivax* ENT family putative nucleoside transporters (TvxNT1–4) and TcoAT1 were PCR-amplified from genomic DNA, cloned into the pNUS-HcN vector, and transfected into SUPKO as described [72]. We observed that SUPKO and all of the transgenic lines generated displayed identical growth relative to the parental LmexCas9 cell line, but that LmexCas9 reached a statistically significant higher cell density (two-tailed *t*-test, *p* < 0.05) (Figure 2). There were differences in growth rate or cell density between SUPKO and the cells expressing the *T. congolense* or *T. vivax* ENT transporters. This can be explained by the possibility that none of these transporters carried sufficient nucleosides to make up for the loss of the *L. mexicana* nucleoside transporters NT1.1, NT1.2 and NT2.

#### 2.2.2. TcoAT1 Basic Characterization and Adenosine Binding Model

**Figure 3:**
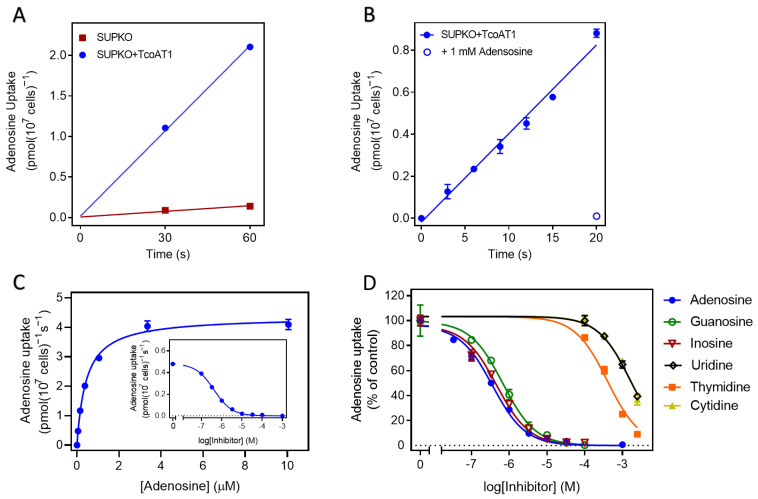
Uptake of 0.05 µM [^3^H]-adenosine in *L. mexicana* SUPKO expressing TcoAT1. (**A**) Uptake in *L. mexicana* SUPKO and SUPKO+TcoAT1 over 60 s. The expression of TcoAT1 restored adenosine uptake in SUPKO. Data represent averages ± SEM of triplicate determinations. (**B**) Uptake of [^3^H]-adenosine in *L. mexicana* SUPKO+TcoAT1 within linear phase of 20 s. (**C**) Adenosine uptake by SUPKO+TcoAT1 in the presence of 0–1 mM unlabeled adenosine. Graph shows one representative out of three independent experiments carried out in triplicate all showing a similar result. (**D**). Substrate specificity of TcoAT1 determined by uptake assays. TcoAT1 has high affinity for both amino- and oxopurines. Inhibition constants (K_i_) for purines were lower than those of pyrimidines. Adenosine uptake is presented as percentage of no-inhibitor control. Figure shows one representative out of three independent experiments carried out in triplicate (% adenosine uptake average ± SEM).

The expression of TcoAT1 in SUPKO restored the uptake of [^3^H]-adenosine. SUPKO+TcoAT1 strongly increased the rate of uptake of [^3^H]-adenosine over 60 s relative to SUPKO (0.035 ± 0.001 vs. 2.3 × 10^−3^ ± 3.5 × 10^−4^ pmol(10^7^ cells)^−1^s^−1^ (*p* = 0.0017, F-test) (Figure 3A). The uptake of [^3^H]-adenosine in SUPKO was not significantly different from zero (*p* = 0.10, F-test), whereas uptake in SUPKO+TcoAT1 was significant (*p* = 0.018) and linear (not significantly non-linear, *p* > 0.99, runs test). In a separate experiment, linearity of adenosine uptake in SUPKO+TcoAT1 was tested with more points over 20 s. Again, there was no significant deviation from linearity (*p* = 0.20) and the rate was highly similar to the first experiment (0.012 ± 0.002 pmol(10^7^ cells)^−1^s^−1^); the addition of 1 mM of unlabeled adenosine completely inhibited [^3^H]-adenosine uptake, indicating a complete saturation of the transporter (Figure 3B).

Using 10 s incubation, being well inside the linear phase of uptake, the affinity of various purines and pyrimidines to the transporter was determined. These kinetic studies revealed TcoAT1 to be a high affinity purine transporter with specificity for adenosine, inosine and guanosine (Figure 3C,D) with a K_m_ for adenosine of 0.42 ± 0.03 µM (Figure 3C, Table 3), statistically identical to the value obtained for adenosine uptake in *T. congolense* bloodstream forms (*p* = 0.30). Also consistent with the transport results in *T. congolense*, this transporter discriminates against pyrimidine nucleosides. Furthermore, TcoAT1, like *T. b. brucei* P1 [44], displayed low affinity for purine nucleobases. This suggests that both the ribose sugar and the purine ring are important for the interaction of this transporter with its substrates.

**Figure 4:**
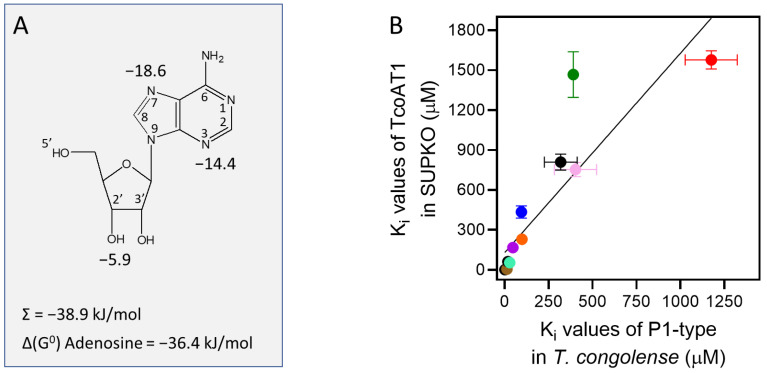
Model of adenosine uptake by TcoAT1. (**A**) Model for the interactions between adenosine and the TcoAT1 binding pocket, showing interactions at N3, N7 and 3′-OH, with Gibbs free energy of interaction indicated in kJ/mol. The sum of these energies and the experimental value for the adenosine ΔG^0^ is also indicated. (**B**) Correlation of K_i_ values for nucleosides and nucleobases in *T. congolense* TcoAT1-type and TcoAT1 expressed in SUPKO. The K_i_ values obtained for each substrate revealed a similar affinity trend for TcoAT1 in the parasite and when expressed in the SUPKO surrogate (r^2^ = 0.78). Slope was significantly different from zero (*p* < 0.0001 by F-test).

To determine the functional groups of the ribose moiety that contribute positively to interactions with the TcoAT1 binding site, the 2′-, 3′- and 5′-hydroxy groups were investigated. While both 2′-deoxyadenosine and 5′-deoxyadenosine did not show a significant difference in affinity compared to adenosine (*p* > 0.4), 3′-deoxyadenosine (cordycepin) presented a 10-fold difference in activity compared to adenosine (K_i_ 4.59 ± 0.63 μM, *p* = 0.006), corresponding to a loss of 5.94 kJ/mol in the Gibbs free energy of binding (ΔG^0^) (Table 3). However, 2′,3′-dideoxyadenosine and adenine presented even lower affinity, with K_i_ values of 51.9 ± 2.6 µM and 761± 103 µM, respectively. These observations, taken together, reveal that the 3′-oxygen is the most important functional group of ribose for recognition by the transporter. In its absence, it appears that some of the binding is taken over by the 2′-hydroxy, indicating a degree of flexibility. In addition, hypoxanthine and 2′,3′-dideoxyinosine showed a lower affinity compared to inosine. These observations are all consistent with a hydrogen bond of ~12 kJ/mol between the TcoAT1 substrate binding site and the ribose moiety, mostly through 3′-hydroxy, with a possible assist from the 2′ position. The main interactions with the purine ring occur at N3 and N7, given the large loss of interaction energy for 3-deazaadenosine (14.4 kJ/mol) and tubercidin (18.6 kJ/mol), respectively. The 6-position and N1 do not seem to contribute significantly to binding, as shown by the pairwise comparisons of adenosine vs. inosine (*p* = 0.31) and adenosine vs. 1-deazaadenosine (*p* = 0.25). These observations result in the binding model for adenosine by TcoAT1 depicted in Figure 4A.

Although uptake assays in *T. congolense* IL3000 and in SUPKO+TcoAT1 did not produce exactly identical K_i_ values for most of the substrates, the pattern of affinity across the inhibitors was comparable in both cell lines. On average, the K_i_ value is consistently ~2-fold higher in TcoAT1 expressed in SUPKO than measured in *T. congolense* IL3000; in a correlation plot the slope was 1.98 (r^2^ = 0.78; Figure 4B). This small difference is likely owing to a difference in the lipid composition of the plasma membrane of *L. mexicana* and of *T. congolense* bloodstream forms. When combined with the Hill scope for these assays being close to −1, as well as the phylogenetic evidence, this greatly strengthens the hypothesis that TcoAT1 is the principal purine nucleoside transporter in *T. congolense*—at a minimum for adenosine. Pyrimidine uptake in *T. brucei* is mostly in the form of uracil [73,74] rather than nucleosides but this is yet to be investigated in *T. congolense*.

#### 2.2.3. TvxNT3 Basic Characterization and Adenosine Binding Model

**Figure 5:**
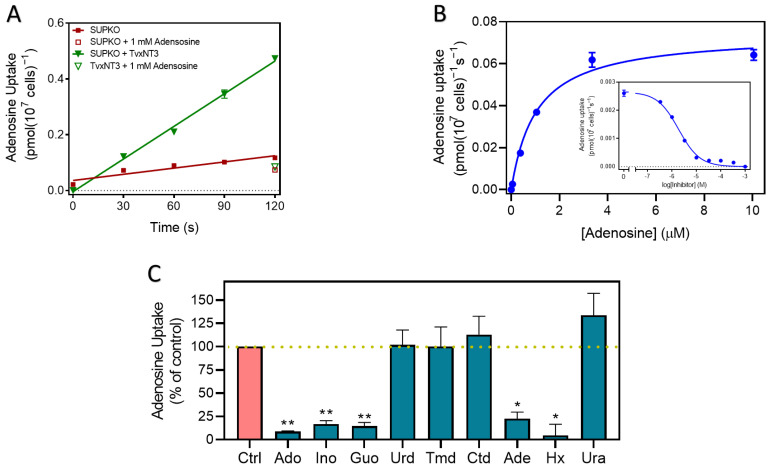
[^3^H]-adenosine (0.05 µM) uptake by TvxNT3 expressed in SUPKO. (**A**) Uptake over 120 s compared to control, and (**B**) Uptake in the presence of 0–1 mM unlabeled adenosine. (**C**) Adenosine uptake in SUPKO+TvxNT3 in the presence of unlabeled nucleosides and nucleobases. Purine and pyrimidine nucleobases and nucleosides at 100 µM competed with 0.05 µM of [^3^H]-adenosine for transport through TvxNT3. Figure shows the average of three independent experiments, each carried out in triplicate (% adenosine uptake average ± SEM, n = 3). Statistically significant difference from the control was determined by an unpaired, two-tailed *t*-test; *, *p* < 0.05; **, *p* < 0.01.

Following the incubation of SUPKO clones expressing each of the four *T. vivax* putative nucleoside transporters with [^3^H]-adenosine, only clones carrying *T. vivax* NT3 (SUPKO+TvxNT3) showed a level of uptake of 50 nM adenosine that was significantly higher than the untransfected SUPKO control (3.9 × 10^−3^ vs. 7.4 × 10^−4^ pmol(10^7^ cells)^−1^s^−1^; *p* < 0.0001, F-test; Figure 5A); uptake was linear for 120 s. Further screening with other nucleosides (100 nM [^3^H]-guanosine, 25 nM [^3^H]-cytidine, 100 nM [^3^H]-thymidine and 100 nM [^3^H]-uridine) did not reveal any differences in uptake between untransfected SUPKO and SUPKO transfected with the other three *T. vivax* NTs either (*p* > 0.05) and we thus proceeded with the detailed characterization of TvxNT3, using [^3^H]-adenosine as the probe.

Figure 5B shows a dose-dependent inhibition of 50 nM [^3^H]-adenosine uptake by unlabeled adenosine, showing complete saturation at high concentrations; conversion to a Michaelis-Menten plot yielded a K_m_ of 1.41 ± 0.35 µM (n = 3).

SUPKO + TvxNT3 cells were incubated with 50 nM [^3^H]-adenosine in the presence of 100 µM of pyrimidine and purine nucleosides and nucleobases to determine the transporter’s substrate specificity. Out of these compounds, adenosine, inosine, guanosine, adenine and hypoxanthine inhibited the uptake of [^3^H]-adenosine significantly (n = 3; *p* < 0.05 or *p* < 0.01), and to less than 25% of the uninhibited control (Figure 5C). This indicates that TvxNT3 exhibits broad specificity for purine nucleosides and nucleobases but not for pyrimidines. The dose-dependent inhibitor assays of adenosine uptake were then carried out using 90 s incubation, which is within the linear phase of adenosine uptake for this transporter (Figure 5A). This allowed for the determination of the inhibition constant (K_i_) of various purine and pyrimidines for this transporter.

The purine ring was the main site of interaction with the transporter binding pocket, as purine bases and nucleosides both displayed high affinity with K_i_ values for inosine, guanosine, adenine and hypoxanthine all below 3 µM, whereas pyrimidine nucleosides uridine and thymidine had orders of magnitude lower affinity (Table 4). Among purine nucleosides, inosine exhibited an approximately 7.7-fold lower K_i_ value for the transporter, than adenosine and guanosine.

The high affinity to TvxNT3 shown by hypoxanthine and adenine appears to suggests that high affinity for this transporter is not dependent on the ribose sugar, unlike the *T. brucei* and *T. congolense* P1 transporters, but similar to P2 [44]. Yet, while the removal of 2’-OH and 5’-OH from adenosine did not affect its affinity, the removal of 3’-OH caused a 6-fold decrease in affinity, corresponding to a loss of 4.47 kJ/mol in the Gibbs free energy of interaction. This clearly shows that 3′-OH does in fact positively contribute to the binding of purine nucleosides, as it does for the *T. b. brucei* P1 transporter [44] and TcoAT1. Moreover, the energy difference δ(ΔG^0^) between inosine and hypoxanthine was determined to be 5.61 kJ/mol and the K_i_ values were highly significantly different (*p* = 0.0021)—similarly indicating a contribution of ribose in the binding of inosine. In contrast, the δ(ΔG^0^) between adenosine and adenine was only 1.34 kJ/mol and the K_i_ values were not significantly different (*p* = 0.17). The conclusion of these seemingly contradictory results must be that nucleosides bind differently in the TvxNT3 binding pocket from nucleobases. Adenine in particular clearly makes either additional interactions or perhaps stronger interactions than the adenine moiety of adenosine. This situation is similar to that recently shown for the *Toxoplasma gondii* transporter Tg244440 [28].

**Figure 6:**
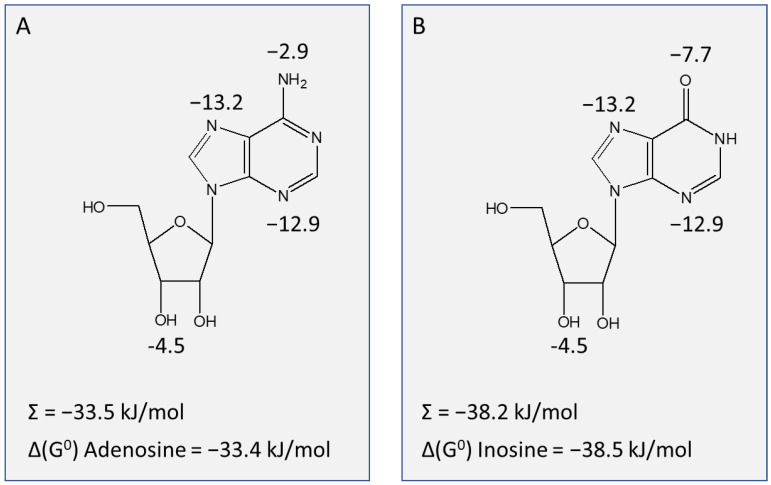
Binding model for TvxNT3 showing points of interaction in adenosine (**A**) and inosine (**B**), with the Gibbs free energy of the interaction indicated in kJ/mol. The sum of the interactions and the experimental interaction energies are also indicated.

We investigated the interactions of the purine nucleosides with TvxNT3, in order to delineate the limitations of modifications that can made while retaining uptake through this transporter. The importance of N7 for binding followed from the low affinity displayed for tubercidin (7-deazaadenosine; δ(ΔG^0^) = 13.2 kJ/mol relative to adenosine). A strong interaction was also evidenced for N3, by the δ(ΔG^0^) of 12.9 kJ/mol between inosine and 3-deazainosine (Table 4). It appears that the 6-amine group of adenosine makes at best a minor contribution (δ(ΔG^0^) = 2.9 kJ/mol, adenosine vs. nebularine) and that the significant difference (*p* = 0.045) in affinity between adenosine and inosine is the result of a positive contribution of the 6-oxo group (δ(ΔG^0^) = 7.7 kJ/mol, inosine vs. 6-thioinosine; *p* = 0.016). These data suggest that the nucleoside binding mode is not dissimilar to the known binding mode of *T. brucei* P1, especially N3, N7, 3′-OH being the main contact points (Al-Salabi et al., 2007), and the sum of the proposed interactions closely matches that of the observed ΔG^0^ for adenosine (Figure 6A). However, inosine is bound slightly different from *T. b. brucei* P1 in that there is a significant interaction with 6-oxo; for this nucleoside too, the sum of the interactions (N3, 6-oxo, N7 and 3′-hydroxy) closely matches the experimental value calculated from the K_m_ (Figure 6B).

### 2.3. P1 Transporters Do Not Transport Any of the Trypanocides in Current Clinical Use

**Figure 7:**
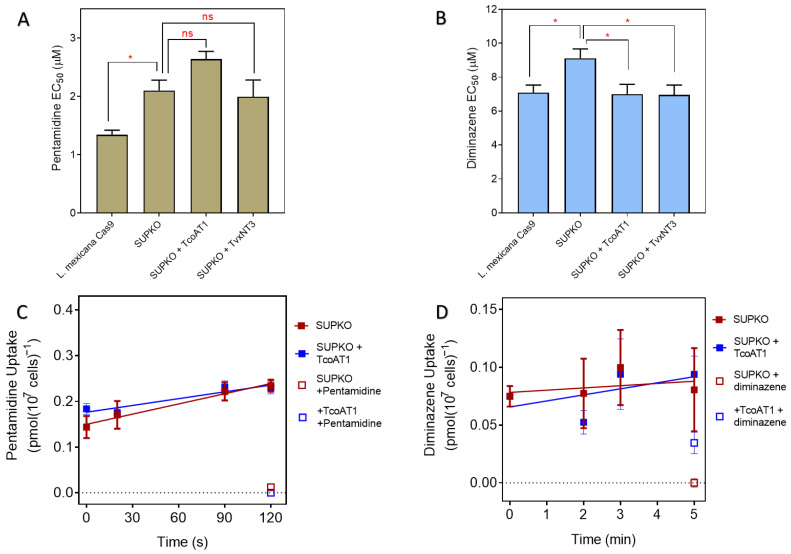
Effect of expression of *T. congolense* and *T. vivax* nucleoside transporters on the activity and uptake of trypanocides in *L. mexicana*. (**A**) Pentamidine sensitivity and (**B**) Diminazene sensitivity. Sensitivity is represented as EC_50_ averages of 4 independent determinations (mean ± SEM). * *p* < 0.05; ns = not significant by unpaired Student’s *t*-test. Uptake of (**C**) 25 nM [^3^H]-Pentamidine or (**D**) 50 nM [^3^H]- in SUPKO+TcoAT1 and SUPKO control cells. At each predetermined time point, uptake was stopped in the corresponding sample by the addition of high concentration of unlabeled substrate followed by centrifugation. Graph was plotted using the average uptake (mean ± SEM) in pmol(10^7^ cells)^−1^) of an experiment performed in triplicate. Lines were calculated by linear regression.

We previously reported that the expression of TcoAT1 in multidrug-resistant *T. brucei* B48 did not result in an increase in sensitivity to diminazene and pentamidine [52]. To confirm this important observation and to investigate whether *T. vivax* NT3 has any role in sensitivity or resistance to the trypanocides in use, we carried out drug sensitivity and uptake assays in *L. mexicana* Cas9, SUPKO, SUPKO+TcoAT1 and the SUPKO+TvxNT3 cell lines in parallel. While SUPKO is slightly less sensitive to pentamidine (1.57-fold) compared to the parental line *L. mexicana* Cas9, the expression of either TcoAT1 or TvxNT3 in SUPKO did not result in a significant increase in sensitivity to pentamidine (Figure 7A), although the expression of the *T. b. brucei* pentamidine transporter TbAQP2 in *L. mexicana* has been shown to sensitize these cells 40-fold to pentamidine [75]. In addition, SUPKO showed only a 1.27-fold decrease in sensitivity to diminazene compared to *L. mexicana* Cas9 (Figure 7B). Expression of TcoAT1 or TvxNT3 in SUPKO did not sensitize the cells to pentamidine and only marginally increased sensitivity diminazene, effectively reversing the minor difference between SUPKO and the parental Cas9 cells (Figure 7A,B). The EC_50_ values of two other trypanocides, melarsomine and suramin, to which *Leishmania* spp are not normally sensitive, remained >100 µM in all the cell lines investigated. Similar to pentamidine, melarsomine sensitivity increased greatly upon expression of TbAQP2 [75].

We next investigated the rate of uptake of [^3^H]-Pentamidine (Figure 7C) and [^3^H]-diminazene (Figure 7D) between SUPKO and SUPKO+TcoAT1. The slopes and intercepts of SUPKO and of SUPKO+TcoAT1 were not significantly different (*p* > 0.05), indicating that the expression of TcoAT1 had no effect on the uptake of either trypanocide. This supports the previous evidence that, unlike P2, P1-type nucleoside transporters are not carriers of currently used trypanocides of the diamidine and melaminophenyl arsenical classes [52,60] and can be targeted as conduits for new drugs without a risk of transport-related cross-resistance [38,65].

### 2.4. Trypanosomal P1 as Carrier of Novel Nucleoside Drugs

#### 2.4.1. Affinity of Nucleoside Drugs to *T. congolense* Nucleoside Transporters

**Figure 8:**
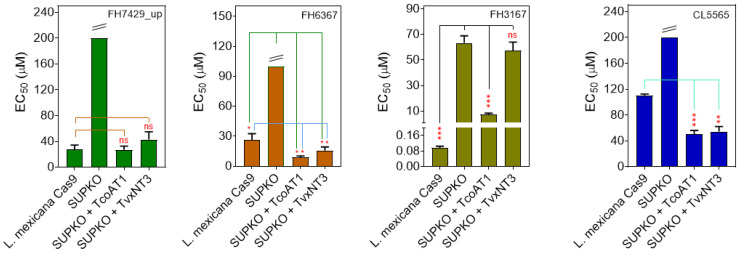
The sensitivity of *T. congolense* and *T. vivax* nucleoside transporters to nucleoside analogs. The expression of TcoAT1 and TvxNT3 in SUPKO restored sensitivity to tubercidin analogs. Sensitivity is represented as EC_50_ averages of three independent determinations (mean ± SEM). * *p* < 0.05; ** *p* < 0.01; *** *p* < 0.001; ns = not significant by unpaired Student’s *t*-test compared with SUPKO. The double line above some bars indicates that the true value could not be extrapolated beyond 250 µM.

The potential of the *T. brucei* P1 nucleoside transporter as a conduit for toxic nucleoside drugs has been described [38]. Therefore, we tested some nucleoside analogs against *L. mexicana* Cas9, SUPKO, SUPKO+TcoAT1 and SUPKO+TvxNT3 in parallel. The CRISPR/Cas9 deletion of nucleoside transporters in *L. mexicana* resulted in significant reduction in the sensitivity of the resultant SUPKO line to tubercidin analogs (Figure 8; Table 5). Notably, SUPKO exhibited ~660-fold resistance to FH3167 (7-deaza-7-fluoroadenosine) compared to parental *L. mexicana* Cas9 cell line, presumably due to the deletion of the LmexNT1.1 and NT1.2 transporters. Similarly, SUPKO lost sensitivity to FH7429_u (7-bromo-7-deaza-3′-deoxyadenosine) to above the 250 µM cutoff, and also displayed significant resistance to FH6367 (7-trifluoromethyl-7-deazaadenosine) (Figure 8). The expression of either TcoAT1 or TvxNT3 in SUPKO restored the lost sensitivity to most of the 7-substituted tubercidin analogs, but not to tubercidin itself (Table 5), which we have above shown to be a poor substrate for both TcoAT1 and TvxN3. The degree to which the sensitivity was increased upon expression of the *T. congolense* and *T. vivax* adenosine transporters differed from partial restoration (e.g., FH3167) to a significantly higher level of sensitivity than Cas9 (e.g., FH6367), presumably reflecting the relative efficiency of transport by the *L. mexicana* and *Trypanosoma* transporters. On this basis it appears that TcoAT1 and TvxNT3 prefer trifluoromethyl and the larger halogens (I > Br > Cl > F) on position 7 of tubercidin. Aromatic substitutions at this position (2-pyridine; TH1008) do not seem to be tolerated.

For the 3′-deoxytubercidins, a similar pattern was observed with respect to the 7-position substitutions. The unsubstituted analog (FH7429_d) displayed only a marginal (TcoAT1) or no measurable sensitization (TvxNT3) upon expression of the transporters. In contrast, the *Trypanosoma* transporters sensitized 7-iodo analog (FH8496) beyond Cas9, the 7-bromo (FH7429_u) and 7-ethyn (FH8505) displayed the same EC_50_ as Cas9 but the 7-fluoro (FH8517) had higher EC_50_ values than Cas9. These results show how the side-by-side comparison of nucleoside analog activity against cell lines that are identical, except for the expression of specific nucleoside transporters, can quickly lead to insights into the SAR of what these carriers may take up.

In order to strengthen this conclusion, we next explored whether the same pattern holds for direct measurements of transporter inhibition constants K_i_ and interaction energy ΔG^0^. In *T. congolense* IL3000, the tubercidin analogs competed with adenosine for uptake through the transporter, hypothesized to be TcoAT1, in a dose-dependent manner. It was observed that, of the 3′-deoxytubercidins, the 7-F and unsubstituted analogs displayed a lower affinity for the *T. congolense* adenosine transporter than the 7-I, 7-Cl and 7-ethene analogs (Table 6). However, the values in terms of Gibbs free energy of interaction were within ~4 kJ/mol of each other and it must be remembered that the K_i_ value is an inhibition constant and does not necessarily inform on uptake rates: a compound may display high binding affinity as an inhibitor without being transported at all. However, compounds that showed the highest activity against the whole parasites, such as FH8470 (7-chloro-7-deaza-3′-deoxyadenosine), also typically exhibited higher affinity to the transporter compared to tubercidin (Table 6). The K_i_ values for TcoAT1 expressed in SUPKO were similar and followed the same ranking as the adenosine uptake in IL3000 bloodstream forms, further supporting the hypothesis that TcoAT1 is the main, and apparently only, adenosine transporter in *T. congolense*. A further set of analogs was then tested on TcoAT1 expressed in SUPKO to further build out the SAR. TH1012 (7-phenyltubercidin), a powerful lead compound against *Trichomonas vaginalis* [68], displayed low affinity to TcoAT1, with a K_i_ value of 146 ± 58 µM. Interestingly, 6-methoxy-7-deazapurine riboside (CL4510) displayed strong activity against *L. mexicana* Cas9 in that assay, which was largely retained (EC_50_ = 2.3 µM) in SUPKO and not changed by the expression of TcoAT1 or TvxNT3. This raises the intriguing possibility that this analog, and possibly others, may be taken up by kinetoplastid cells independent from the known nucleoside transporters.

#### 2.4.2. Affinity of Nucleoside Drugs to *T. vivax* Nucleoside Transporters

Similar to the P1-type transporters in *T. brucei* and *T. congolense*, TvxNT3 showed a preference for 7-bromo-3′-deoxy-tubercidin (FH 7429_u) over the 7-unsubstituted analog (FH 7429_d) although the affinity for both was quite low (Table 7). A difference with TcoAT1, however, was that the replacement of 6-amine with OMe (CL4510) lowered the K_i_ for tubercidin by an order of magnitude, consistent with our conclusion that position 6 contributes positively to the interaction with TvxNT3 (Figure 6) but not TcoAT1 (Figure 4).

#### 2.4.3. Antitrypanosomal Activity of Nucleoside Analogs In Vitro

A series of nucleoside analogs was screened for activity against BSF *T. congolense* IL3000 and the diminazene-resistant *T. congolense* strain 6C3 to determine the potential for cross-resistance with diminazene aceturate [60], *T. b. brucei* clone B48—all expressing only P1-type adenosine transporters [76]—in addition to *T. evansi* and *T. equiperdum*. EC_50_ values for these strains and species are displayed in Table 8, alongside EC_50_ values for *T. vivax* obtained from Mabille et al. [69]. Clearly, the 7-halogenated-3′-deoxytubercidins had highly potent activity against all the AAT trypanosomes: Br > I ~ Cl > F. It was also observed that 3′-deoxytubercidin was a more potent agent against *T. b. brucei* B48 and *T. congolense* than tubercidin (17.2-fold and 150-fold, respectively).

**Figure 9:**
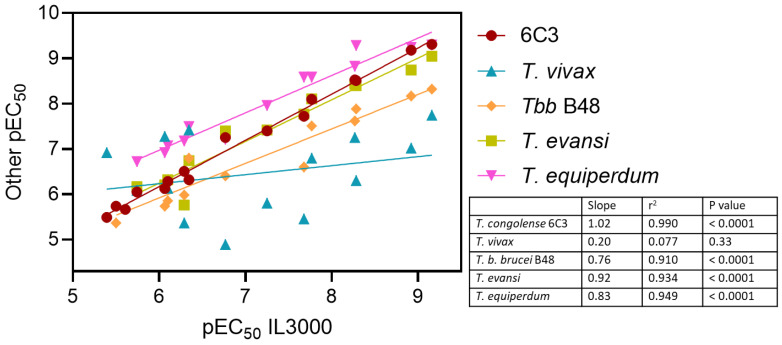
Correlation between the −^10^log(EC_50_) (pEC_50_) of adenosine antimetabolites against *T. congolense* IL3000 with the pEC_50_ for other *Trypanosoma* cell lines or species. Lines were obtained by linear regression and slopes, correlation coefficients and *p* value for slope being non-zero were calculated by GraphPad Prism v8.

The comparison between *T. congolense* strains IL3000 and 6C3 shows that most compounds displayed high activity with no cross-resistance to diminazene (Table 8). Indeed, a correlation plot of pEC_50_ values for these strains yielded an r^2^ of 0.99 and a slope of 1.020 (significantly non-zero, *p* < 0.0001, F-test), i.e., a near perfect agreement between the datasets (Figure 9). Although we have previously shown that tubercidin is dependent on the P2 transporter for activity against *T. b. brucei*, and therefore cross-resistant with pentamidine [46,48], the 7-halotubercidins and 7-halo-3′-deoxytubercidins are not [38].

A similar SAR pattern was also observed for *T. b. brucei* B48, *T. evansi* and *T. equiperdum*, likewise yielding strong correlations with the IL3000 pEC_50_s (all r^2^ > 0.90; Figure 9). In contrast, using the dataset of Table 8, there was a poor correlation with the pEC_50_s of *T. vivax* (r^2^ = 0.077; slope 0.199 ± 0.198, not significantly different from zero, *p* = 0.34)—this despite the adenosine transporters of *T. vivax* and *T. congolense* being quite similar (Section 2.2). A closer look reveals that 3′-deoxytubercidin analogs with larger N7 substitutions (FH8505, FH10659, TH1008), or none (FH7429_d) performed particularly poorly against *T. vivax* (10–165-fold compared to *T. congolense*). The halogenated 3′-deoxytubercidins did display mid-nanomolar EC_50_s against *T. vivax*, but the values were still 10–96-fold higher than for *T. congolense* and similar trends were in evidence relative to *T. b. brucei*, *T. evansi* and *T. equiperdum* and it appears that 3′-deoxytubercidins are generally less active against *T. vivax* than against other African trypanosome species. Indeed, whereas the 7-fluoro and 7-chloro-3′-deoxytubercidin were much more active than the equivalent tubercidin with intact ribose against all AAT trypanosome species, the reverse was the case for *T. vivax*. However, the excellent activity of 7-halo-3′-deoxytubercidins against *T. evansi* and *T. equiperdum* (Table 8) makes them highly promising leads for the treatment of non-tsetse transmitted animal trypanosomiasis, specifically surra and dourine.

**Figure 10:**
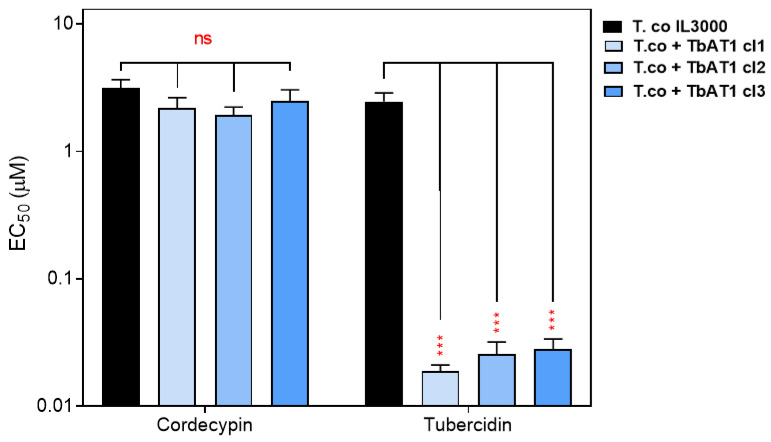
Effect of expression of TbAT1 on the sensitivity of *T. congolense* to tubercidin. Sensitivity is represented as average EC_50_ (mean ± standard error of mean) of three independent determinations. *** *p* < 0.001; ns = not significant by unpaired Student’s *t*-test.

The P2/TbAT1 transporter mediates the uptake of multiple trypanocides, but this transporter is lost in many drug-resistant strains [20,43,50,51,77]. Thus, nucleoside analogs that do not utilize this transporter have higher potential for development as new drugs that replace the ones for which resistance has already developed. This prompted us to determine whether P2/AT1 is involved in the uptake of the 6-position-modified 7-deazapurineriboside analogs, using a *T. congolense* IL3000-based cell line expressing *TbAT1* [18]. Standard resazurin-based drug sensitivity assays showed that as expected, TbAT1 expression caused a highly significant sensitization to tubercidin (*p* < 0.0001) in three independent clones (Figure 10), as tubercidin is a good substrate of TbAT1/P2 [46] but at best a poor substrate for the *T. congolense* adenosine transporter, TcoAT1 (Table 6) and as a result tubercidin is a relatively poor agent against *T. congolense* (Table 8).

#### 2.4.4. The Effect of Tubercidin Analogs on Growth, Cell Cycle and Morphology of *T. congolense*

**Figure 11:**
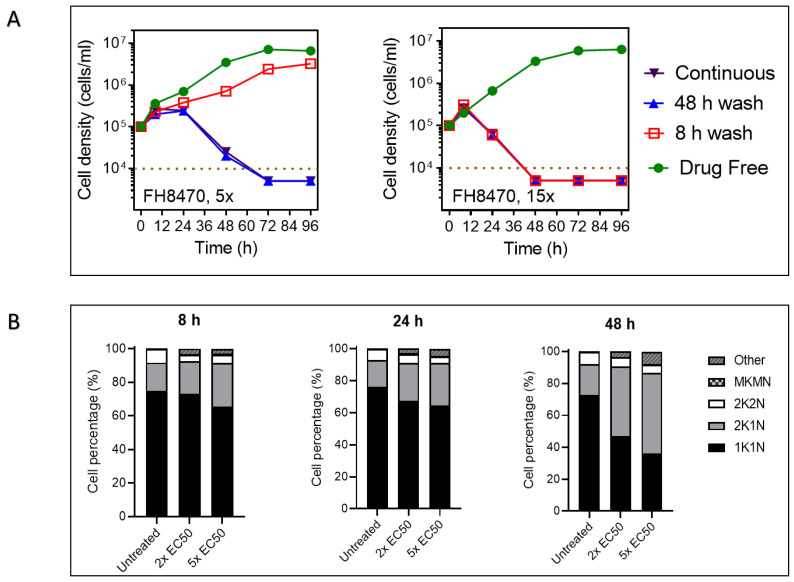

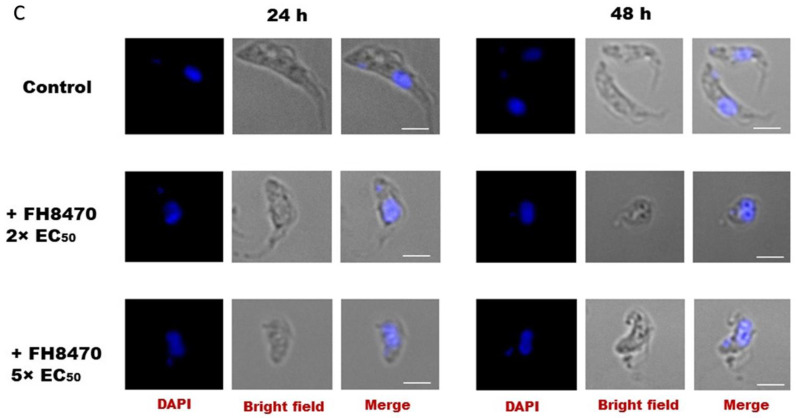
The effect of tubercidin analog FH8470 on growth, cell cycle and morphology of *T. congolense.* (**A**) Manual cell count of cultures grown in the presence or absence of 5 × or 15 × EC_50_ of tubercidin analog FH8470. For short exposure, cultures were washed through centrifugation and resuspended in fresh medium. The dotted brown line indicates the detection limit, being 10^4^ cells/mL. For the purpose of this graphical representation, where no cells were observed in the counting chamber, the value of 5 × 10^3^ or 4 × 10^3^ was entered. (**B**) Effect of nucleoside analog FH8470 on the cell cycle progression in *T. congolense*. Cells were seeded at 10^5^ cells/mL in growth medium with or without test drug at the desired concentration. At each predetermined period, approximately 10^6^ cells were harvested, transferred to a glass slide and fixed. The slide was then DAPI-treated and covered with a coverslip, and images were acquired using Olympus IX71 DeltaVision Core System fluorescent microscope (Applied Precision, GE, Rača, Slovakia). The configurations of nucleus and kinetoplast for at least 300 cells were observed in each group; 1K1N = one kinetoplast and one nucleus; 2K1N = two kinetoplasts and two nuclei; 2K2N = two kinetoplasts and two nuclei; MKMN = multiple kinetoplasts and/or nuclei; other = other aberrations such as no nucleus or no kinetoplast. Graphs show percentage mean ± SEM of three independent determinations. (**C**) Images of *T. congolense* cells exposed to nucleoside analog FH8470 for 24 h or 48 h. Test drug at desired concentration was added to BSF *T. congolense* culture followed by incubation for 24 h or 48 h. Cells were harvested at predetermined point, applied to a glass slide, fixed and treated with DAPI before the slide was covered with a coverslip. Images were acquired using a Delta Vision Core fluorescent microscope and analyzed using ImageJ software. Treated culture showed higher percentage of cells with conjoined nuclei and two kinetoplasts, often accompanied by mottled cell surface and loss of slender BSF shape. White bar represents 3 µM.

The effects of short and long exposure to a representative tubercidin analog on the growth of BSF *T. congolense* were investigated as described previously [38]. A short exposure (8 h) of *T. congolense* to a concentration of 15 × EC_50_ FH8470 resulted in the death of the cells within 48 h, whether the drug was continuously present or washed out. On the other hand, exposure for 48 h or continuously to 5 × EC_50_ FH8470 killed the parasites within 72 h (Figure 11A, right panel). Clearly, the compound, at that concentration, induces irreversible damage to the cells which causes them to die later. No live trypanosomes were recovered at the 96 h point (i.e., 88 h after the removal of the compound) and it thus appears that these compounds are trypanocidal. Exposure to a lower concentration of 5 × EC_50_ showed a similar pattern but required a longer exposure time before the effect became irreversible (Figure 11A, left panel).

The nucleus and kinetoplast configuration of a trypanosomatid cell reflect the cell cycle phase at that point in time [78,79]. Therefore, the effect of a test drug on cell cycle progression can be investigated by comparing the DNA content/configuration of drug-exposed *Trypanosoma* cell population with that of an untreated control [80,81]. In an untreated *T. congolense* IL3000 BSF culture, we observed that 70–80% of the cells present with a single kinetoplast and nucleus throughout the initial lag and logarithmic growth phases, which indicates a preparatory or early G1 phase of cell cycle (Figure 11B). A further 15–20% of the cells bear two kinetoplasts and a single nucleus (2K1N) while 5–8% show a two kinetoplasts and two nuclei configuration (2K2N) representing the G1/G2 and M phase of the cell cycle, respectively; this is a normal distribution for log phase trypanosomes [82]. The addition of 2× or 5× EC_50_ of FH8470 to a *T. congolense* culture resulted in a progressive decrease in the percentage of cells at 1K1N, and an increase in the percentage of cells at 2K1N DNA configuration (Figure 11B). As early as at the 8 h mark, the number of cells with 2N2K configuration was reduced by 40–50% of untreated control and this remained depressed throughout. These data strongly suggest that FH8470 arrests cell cycle progression, most likely through the inhibition of nuclear DNA synthesis and/or mitosis.

Several cells at 24 h and 48 h post-treatment with either 2× or 5× EC_50_ of FH8470 presented with two kinetoplasts and conjoined nuclei, indicating failure to complete nuclear division. Such nuclei have typically lost the usual round shape and appeared larger than the nuclei in cells bearing a single kinetoplast. In addition, a large percentage of the cells exposed to FH8470 have lost the slender BSF shape and had a mottled cellular membrane arising from either direct damage, or perhaps from pressure exerted by the larger conjoined nucleus (Figure 11C).

### 2.5. Toxicity of Tubercidin Analogs on Mammalian Cells

The use of the resazurin assay to investigate the toxicity of test compounds on mammalian cells and to quantify their selectivity for parasite cells has been reported previously [83]. Most of the assayed tubercidin analogs exhibited low toxicity against human embryonic kidney (HEK) cells (Table 9), which translated into high selectivity to the parasites. We hypothesize that this is likely because their entry into the cell, through high affinity adenosine transporters with broad specificity, is lacking in mammalian cells, which feature carriers with much lower affinity [26,37]. Alternatively, the enzymes of purine metabolism in mammalian cells may not activate these nucleoside prodrugs, as known for allopurinol and formycin B, which derive selective toxicity at not being processed to the AMP analog in mammalian cells [84]. Notably, the 3′-deoxytubercidin analogs showed the highest selectivity for parasites, especially FH7429_u, FH7429_d, FH8470 and FH8496 (Table 9). In contrast, FH6367, featuring an intact ribose, showed higher toxicity to HEK cells than to *T. evansi* and *T. congolense*.

## 3. Discussion

The lack of capacity for de novo synthesis of purines by protozoa [25] has resulted in the development of a class of highly specialized and highly efficient membrane transporters for the internalization of this essential nutrient [26]. However, the three main AAT-causing pathogens in Africa, *T. b. brucei*, *T. congolense* and *T. vivax* have not received the same level of attention from researchers over the past decades. The availability and ease with which *T. b. brucei* could be cultured and manipulated have resulted in an over-reliance on this one species in trypanosomatid research. In contrast, the study of *T. congolense* and *T. vivax* has been limited by the unavailability of cellular and genetic modification tools for a long time, and even more by the challenges of their in vitro culture which even now is limited to a few strains of *T. congolense*, while *T. vivax* can only be maintained briefly ex vivo, but still not cultured. However, the use of the *brucei* model in animal trypanosomiasis research is limited by genetic, pharmacological and metabolic differences between *T. brucei* and *T. congolense* (and presumably *T. vivax*), which are becoming ever more apparent [60,85]. For *T. congolense*, although in vitro axenic culture has been available for some time [86], reliable and functional genetic modification tools have only recently been developed [87]. Unfortunately, long-term axenic culture and culture techniques for *T. vivax* are not yet established and only a few strains reliably achieve infection in mice [88,89,90].

The *T. brucei* nucleoside transporters have been thoroughly studied for three decades. In this species, two types of nucleoside transporters, P1 and P2, have been identified, and their substrate specificity well characterized [34,39,41,46,91]. P1 is a broad-specificity high-affinity purine transporter that interacts with nucleoside substrates through the N3, N7, 3′-OH, and 5′-OH functional groups that are common to all the natural purine nucleosides [40,44]. On the other hand, the P2 transporter is exclusively an aminopurine carrier that interacts through N1, C6-NH_2,_ N9 and π-π stacking of purine ring. The high affinity to the N(1)=C(6)-NH_2_ motif has allowed it to mediate the uptake of drugs such as diamidines, isometamidium and melaminophenyl arsenicals [92,93]). While P1-type transporters are also expressed in other trypanosomatids, the P2 transporter has so far only been found in the *brucei* group trypanosomes. Specifically, *Leishmania* spp. and *T. cruzi* express two P1-type transporters, each with their different kinetic characteristics [27,61].

While the P2 transporter has therefore been very successfully leveraged to deliver chemotherapy, the development of resistance to these old drugs has resulted in the circulation of populations without a functional P2 transporter [7]. The discovery that the P1 rather than P2 is expressed in other pathogenic trypanosomatids [27,52,61] has opened up the possibility for a more effective targeting of the multi-species disease AAT, particularly since no equivalent of P2 is expressed in *T. congolense* or *T. vivax* [53].

Purine metabolism in *T. congolense* and *T. vivax* is not yet fully understood. Earlier studies have pointed out that adenosine cleavage in *T. congolense* depends on purine nucleoside phosphorylase, whereas *T. vivax* and *T. brucei* utilize purine nucleoside hydrolase for that purpose [94]. The *T*. *congolense* BSF culture supernatants showed significant accumulation of guanine and xanthine, likely as the by-products of hydrolysis of guanosine and xanthosine by the parasite hydrolases/nucleosidases [85].

TcoAT1 is closest to *T. brucei* NT10 out of the eight gene variants that code for P1-type transporters in the *T. brucei* genome [52], suggesting it should be a broad specificity purine nucleoside transporter. Conforming to these expectations, we have found TcoAT1 to be a high affinity transporter for adenosine (K_m_ = 0.42 µM), inosine (K_i_ = 0.55 µM) and guanosine (K_i_ = 0.8 µM). In addition, this transporter excludes purine nucleobases and pyrimidines. These findings are consistent with our earlier findings [52] but provide far more detail than was possible in the *T. brucei* B48 expression system and shows the utility of the *L. mexicana* SUPKO cell line as recently shown with the characterization of the *T. vaginalis* ENT3 nucleoside transporter [29]. Considering the essential need of adenosine, guanosine and inosine, it is not surprising that the single transporter expressed in this species mediates the uptake of all three. The earliest studies on nucleoside transport in *T. congolense* showed that excess unlabeled adenosine, inosine, and guanosine could reciprocally and completely inhibit each other’s uptake [95]. A more recent study involving the heterologous expression of *TcoAT1* in the *T. brucei* TbAT1-null clone B48 has shown an increase in the uptake of adenosine and inosine [52]. The TcoAT1 characteristics observed in this study are highly similar to NT10 expressed by the short-stumpy BSF and procyclic forms of *T. brucei*, with K_m_ values of 0.41 µM for adenosine and 0.53 µM for inosine (Table 10) [40] but also quite similar to the BSF P1-type transport [44].

While TcoAT1 and TvxNT3 have large differences in their affinity to nucleobases, they both showed high affinity to adenosine, inosine and guanosine in similar fashion to P1 nucleoside transporters of *T. brucei* [40]. These three transporters similarly interact with nucleoside substrates, with the main contact points being N7, N3 and 2′-OH. *T. brucei* P1 additionally interacts with the 5′-OH, which does not seem to be involved in *T. congolense* and *T. vivax* adenosine transport. 

A further difference is a contribution by the 6-keto group of inosine to binding by TvxNT3 only. The permissibility for nucleobases for TvxNT3 is the result of only a minor contribution from the 2′-OH (−4.5 kJ/mol) relative to the TcoAT1 (~12 kJ/mol) and TbbP1 interactions of 15 and 14 kJ/mol for 2′-OH and 5′-OH, respectively [44]. In addition, purine nucleobases may be able to assume an energetically favorable orientation in the TvxNT3 binding pocket that is different from the nucleoside binding. The high affinity of TvxNT3 for purines is maintained by the extra interaction at position 6 and the two strong hydrogen bonds at N3 and N7. The similarities in binding modes explain that all three carriers function as broad specificity purine-selective nucleoside transporters, while the differences explain some of the disparities regarding specific nucleoside analogs.

The presence of transporters with highly similar characteristics regarding nucleoside binding and permissibility in *T. brucei, T. congolense* and *T. vivax* presents an interesting avenue for drug development. The disparity in innate sensitivity to diamidine and arsenical between *T. brucei* and *T. congolense* has been shown to result from a lack of AT1 and AQP2 transporters in *T. congolense* [18]. We have confirmed that the expression of P1 transporters of *T. congolense* and *T. vivax* does not affect the sensitivity of the currently used drugs diminazene, pentamidine, melarsomine and suramin. This suggests that purine nucleoside analogs can be designed to target the P1 transporters of veterinary trypanosomes with minimal risk of cross-resistance to the current trypanocides. Therefore, we tested the chemotherapeutic potential of the TcoAT1 and TvxNT3 using toxic nucleoside analogs with reported affinity to *T. brucei* P1. It was found that the expression of either TcoAT1 or TvxNT3 in the resistant *L. mexicana* SUPKO sensitized these NT-*null* cells to most of the tubercidin analogs—clear proof of internalization through the expressed heterologous carriers.

The uptake of toxic nucleosides, particularly tubercidin and cordycepin, has to date been thought to be mediated largely by the P2 transporter in *T. brucei*: the TbAT1-KO strain showed 77-fold and 17-fold resistance to tubercidin and cordycepin, respectively [46,47]. In a recent study, a tubercidin resistance factor of 28.7 was reported in TbAT1-KO compared to the wild type (0.15 ± 0.03 vs. 4.3 ± 1.3 μM EC_50_) [38]. In this study, the EC_50_ of tubercidin in *T. congolense*, was determined as 3.16 ± 0.50 μM, similar to *T. brucei* lacking *TbAT1*. *T. b. brucei* B48 exhibits similar sensitivity to tubercidin as *T. congolense*, as both parasites express only a P1-type transporter. Several modifications were carried out on the purine ring and/or the ribose sugar in order to generate tubercidin analogs with improved targeting of the trypanosomal P1 transporter. We have recently reported the structure activity relationship (SAR) of the tubercidin analogs in *T. brucei* [66].

Given the similarity in activity of tubercidin against *T. brucei* B48 or TbAT1-KO and *T. congolense*, we expected that the modification of tubercidin will also yield improved activity against *T. congolense* and *T. vivax*. We investigated the hypothesis through a series of comparative drug sensitivity tests and uptake assays in whole parasites and isolated transporters expressed in surrogates. For this purpose, cell lines expressing adenosine transporters of *T. congolense* (TcoAT1) and *T. vivax* (TvxNT3) in *L. mexicana* NT1+NT2-KO (SUPKO) were utilized. In parallel, the analogs were also tested directly on the organisms, the combination of the two screens, plus the transporter affinity studies, allowing more insightful conclusions on separating issues of transporter permeability and transporter-independent sensitivity of the parasites for the nucleosides tested.

As expected, some tubercidin analogs presented sub-micromolar activity dependent on the expression of TcoAT1 or TvxNT3, with a similar pattern of SAR to *T. brucei* P1 [66]. Of particular note, the incorporation of the cordycepin motif (3′-deoxyadenosine) into tubercidin (7-deazaadenosine) resulted in higher in vitro activity against *T. congolense*, *T. evansi*, *T. equiperdum* and *T. brucei* B48, although it did not result in higher affinity to TbbP1 [38], TcoAT1 and TvxNT3 transporters. The addition of a functional group to the C-7 of tubercidin yielded still higher activity, and greatly improved affinity to the P1 transporters of all the three species. In general, tubercidin analogs bearing the larger halogen groups at C-7 were more active than those containing a fluor or a small unsaturated or an aromatic substituent. Only against *T. vivax* did the 3′-deoxytubercidin analogs display lower activity than the equivalent ribonucleoside, but this cannot be attributed to a difference in adenosine transporter, as TvxNT3 was in fact much less dependent on interactions with the ribose and specifically 3′-OH (δ(ΔG^0^ 4.5 kJ/mol relative to adenosine) than TcoAT1 (δ(ΔG^0^) 5.9 kJ/mol) or TbbP1 (δ(ΔG^0^) 15 kJ/mol [44]), and the expression of TvxNT3 sensitized SUPKO to 7-bromo-3′-deoxytubercidin (FH7429_u). Thus, the relative insensitivity of *T. vivax* to 3′-deoxytubercidins can be confidently attributed to a specific difference with other animal trypanosomes at the level of intracellular target enzymes. Regardless, it should still be noted that the 7-Cl, 7-Br and 7-I-3′-deoxytubercidins were the most potent nucleosides against *T. vivax* identified here, with EC_50_ values between 18 and 96 nM. The low-nM or sub-nM activity of these compounds against *T. evansi* and *T. equiperdum* make them exciting candidates for the treatment of surra and dourine.

Given that the 3′-deoxy modification was unfavorable for *T. vivax*, leading to an unequal level of activity of 3′-deoxytubercidin analogs among the three AAT-causing species, a different modification was carried out. The C-6 amino group of tubercidin was substituted with different groups while ribose remained intact, to yield C-6 alkylated tubercidin analogs. Very recently, 6-methyltubercidin analogs were reported to exert comparable activity against *T. b. rhodesiense, T. cruzi* and *L. infantum* [97,98]. This broad-spectrum antikinetoplastid activity is uncommon and affirms the potential of targeting multiple trypanosomatid species through P1-type nucleoside transporters. Thus, future efforts into discovery of pan-AAT nucleoside drugs should focus on 6-substituted tubercidin analogs. While these analogs were not as active as the 3′-deoxytubercidin analogs in *T. brucei*, *T. congolense*, *T. equiperdum* and *T. evansi,* the multispecies activity they have exhibited at ˂ 0.2 µM EC_50_ makes them more promising candidates to target both AAT and the non-tsetse transmitted animal trypanosomiases (NTTAT). As the areas of endemicity of veterinary trypanosomes increasingly overlap on the global map [5], there is an urgent need to develop drugs that are active against all five *Trypanosoma* species.

An ideal chemotherapeutic agent should be selectively toxic to the target cell while sparing the host cell. Therefore, we tested the toxicity of tubercidin analogs against human embryonic kidney (HEK) cells using the standard Alamar Blue assay. Most of the tubercidin analogs exhibited low activity against HEK cells, which translates into high selectivity indexes (SI). Specifically, the 3′-deoxytubercidin analogs demonstrated very high selectivity to parasites especially FH7429_u, FH7429_d, FH8470 and FH8496. The only exception was FH6367, with an intact ribose, which showed higher toxicity to HEK cells than to *T. evansi* and *T. congolense*. As with almost every other veterinary trypanocide [1], a nucleoside drug against AAT would be injected intramuscular rather than administered orally, which would ensure rapid distribution in the bloodstream without the complications of intestinal absorption, stability in stomach acids or the complications of the ruminant digestive system. We have previously shown that 3′-deoxy-tubercidin is curative in a *T. b. brucei* mouse model, even for the difficult to treat stage, where the parasite has infected the central nervous system [38]. It should be noted that this compound cured the AAT model by both oral and intraperitoneal administration [38].

Widespread resistance to the currently used trypanocides has continued to undermine the vital role chemotherapy plays in the control of animal trypanosomiases [99]. It is therefore important that new drugs neither share similar targets with the currently used trypanocides nor exhibit a potential for cross-resistance. The fact that the modified tubercidin analogs reported here mostly utilize P1-type transporters, and not the multidrug P2 transporter is a welcome development. Nevertheless, we tested the tubercidin analogs against the diminazene-resistant *T. congolense* strain 6C3, which displays reduced mitochondrial membrane potential [60]. There was no significant difference in the sensitivity of 6C3 and the wild type to the tubercidin analogs. Since multiple trypanocides including diminazene, isometamidium, pentamidine and nifurtimox target mitochondria [60,79,100,101], the risk of cross-resistance between the tubercidin analogs and these drugs is low.

To start exploring the mechanism of action of the tubercidin analogs, *T. congolense* culture was exposed to one of the analogs, and the cell growth rate, cell cycle and morphology were examined. We observed that the killing of trypanosomes by low concentrations of these compounds can be reversed or delayed by the removal of drug pressure after short exposure, but the drug action becomes rapidly irreversible at higher concentrations. Furthermore, the addition of 2 × or 5 × EC_50_ of FH8470 to *T. congolense* culture resulted in a progressive decrease in the percentage of cells bearing 1K1N, with a corresponding increase in the percentage of cells at 2K1N DNA configuration and decrease in 2K2N cells. This suggests that FH8470 prevents the cells from completing M phase, thus inhibiting nuclear division. This is corroborated by the appearance of several cells with conjoined nuclei among the cells bearing double kinetoplasts, indicating a failure to divide. Such nuclei typically have lost the usual round shape and appear larger than the nuclei in cells bearing 1K. In a previous study [79], it was reported that >10% of *T. brucei* cells treated with melarsoprol bear conjoined nuclei and double kinetoplasts. Further investigations revealed that the melarsoprol-mediated inhibition of nuclear division is mediated by multiple kinases [79].

In a previous study, RNAi has indicated that tubercidin inhibits multiple enzymes in the glycolytic pathway in *T. brucei* including hexokinase and phosphoglycerate kinase [102]. However, recently, a genome-wide RNAi in *T. b. brucei* identified that six RNAi inserts including those targeting the *TbAT1* transporter and adenosine kinase (ADKIN; Tb927.6.2300) are involved in the action of 3′-deoxytubercidin. Subsequently, the roles of TbAT1 transporter and ADKIN were confirmed, with the targeted knockdown of both genes resulting in significant resistance to 3′-deoxytubercidin [38]. Interestingly, the selected 3′-deoxytubercidin analogs showed metabolic stability in experiments simulating phase I and phase II metabolism, which affirms their potential as therapeutic agents [66].

## 4. Materials and Methods

### 4.1. Cell lines and Culture

The bloodstream form (BSF) of wild-type and mutant parasites belonging to the Trypanozoon subgroup including *T. b. brucei* s427, *T. evansi* AnTat 3/3 and *T. equiperdum* BoTat1 [20] were cultured in supplemented HMI-9 medium and maintained as described [18]. BSF *T. congolense* IL3000 and derived mutants were cultured in TCBSF3 [86] without blood cells, as previously described [81]. Promastigotes of *L. mexicana* Cas9 and derived cell lines were grown as described [29] with *L. mexicana* Cas9 maintained in 32 μg/mL hygromycin while the mutant SUPKO+NT strains were maintained with neomycin G418 at 50 μg/mL ([64]. Human embryonic kidney (HEK) cells were cultured and maintained as reported [83].

### 4.2. Genetic Manipulation of L. mexicana Promastigotes

The information for the *T. congolense* adenosine transporter (TcoAT1) and four putative *T. vivax* nucleoside transporters was obtained in the TritrypDB (tritrypdb.org/tritrypdb) genome database. Primers were designed to flank each gene upstream of the 5′ end (forward) and downstream of the 3′ end (reverse) as described (primer sequences in Supplementary Table S2). Following amplification from the genomic DNA of *T. congolense* or *T. vivax*, DNA for each extracted gene was ligated into the pNUS-HcN expression vector doubly digested with *Nde*I using the NEBuilder kit according to the manufacturer’s instructions (New England Biolabs). Construct was transformed into competent *E. coli* cells, and ligation was confirmed by restriction enzyme digestion of isolated plasmid DNA followed by Sanger sequencing (Source BioScience, Nottingham, UK). Confirmed plasmids were then transfected into *L. mexicana* cell line in which both NT1 and NT2 were doubly knocked out (SUPKO) and derived from *L. mexicana* Cas9 [64] as described [29]. Clones grown in medium containing 50 μg/mL neomycin (G418) were collected for further confirmation by PCR with genomic DNA using a forward primer of the transporter gene and a reverse primer of the plasmid.

### 4.3. Drug Toxicity Assays

The activity of clinical trypanocides and the nucleoside analogs against various cell lines was assessed using a resazurin-based (Alamar blue) drug sensitivity assay to determine the EC_50_ in different cell lines, which also allowed for the investigation of cross-resistance and for toxicity to mammalian cells, exactly as described, in 96-well plates using 11 doubling dilutions of a test compound starting at 100 µM, and a drug-free control [18,103]. EC_50_ values were determined by plotting to an equation for a sigmoid curve with variable slope (GraphPad Prism 8). All values were the average of at least three independent determinations on different days.

For the direct assessment of test compounds on parasite growth, a manual cell count of cultures grown in the presence or absence of test drugs was carried out to determine the effect of test drugs on the multiplication rate of the parasites as described [83]. The cells were adjusted to the required density in 2 mL of the appropriate medium with or without the drug/compound of interest, in a 24-well plate, followed by incubation under standard conditions. The cells were then manually counted at intervals with a Neubauer cell chamber using a phase-contrast light microscope (Axioscope, Zeiss, Jena, Germany).

To assess the effect of the drug on the cellular morphology and configuration of nuclei and kinetoplasts, treated cells were collected after incubation period, fixed, DAPI-treated and viewed under an Olympus IX71 DeltaVision Core System microscope (Applied Precision, GE) using the SoftWoRx suite 2.0 software (Applied Precision, GE). At least 300 cells were scored per slide for the number of kinetoplasts (K) and nuclei (N) as 1N1K, 1N2K, 2N2K, MKMN (M = many) and ‘others’ (aberrant N/K distribution) [81].

### 4.4. Radiolabel Drug and Nucleoside Uptake Assays

Uptake assays using radiolabeled trypanocides and/or nucleosides were carried out as described previously [27,104]. Briefly, parasite cultures were harvested, washed twice in assay buffer (33 mM HEPES, 98 mM NaCl, 4.6 mM KCl, 0.5 mM CaCl_2_, 0.07 mM MgSO_4_, 5.8 mM NaH_3_PO_4_, 0.03 mM MgCl_2_, 23 mM NaHCO_3_, 14 mM D-glucose, pH 7.3), then resuspended and adjusted to a density of 1 × 10^8^ cells/mL in assay buffer. The radiolabeled permeant in the presence or absence of different concentrations of potential inhibitors reconstituted in 100 μL assay buffer was deposited on top of 250 μL oil mix in microcentrifuge tubes; 1 × 10^7^ parasites in 100 μL assay buffer were added to the tube and incubated for a pre-determined interval. The reaction was stopped by the addition of 1 mL of a high concentration of ice-cold unlabeled substrate in assay buffer (‘stop solution’) and centrifugation, or by immediate centrifugation at 145,000 rpm as appropriate. The microcentrifuge tubes were flash-frozen, and the bottom of each tube containing the cell pellet was cut off and transferred to a scintillation vial. Cell pellets were lysed in 2% SDS, after which scintillation fluid (Scintlogic U, Lablogic) was added, followed by overnight incubation in the dark. Scintillation was measured in a 300SL (Hidex) scintillation counter (Hidex). All the assays were conducted in triplicate and analysis was carried out using Microsoft Excel and GraphPad Prism. All kinetic parameters were obtained on at least 3 separate occasions.

The dose-dependent uptake assay allowed for the affinity of nucleoside analogs to different nucleoside transporters to be determined by measuring the extent to which the analogs compete with and inhibit the uptake of radiolabel adenosine [27]. This allowed for the determination of biochemical parameters such as K_m_ and V_max_ through the application of the Michaelis–Menten equation V_0_ = V_max_([substrate]/([substrate] + K_m_)). The 50% Inhibitory Concentration (IC_50_) of the purines and pyrimidines and their analogs was determined from non-linear regression to sigmoid curves with variable slope (Prism 8), and the IC_50_ of the inhibitor and the K_m_ of the labeled permeant were then used to calculate the inhibition constant (K_i_) of the analogs through the Cheng–Prusoff equation: K_i_ = IC_50_/(1 + (*L*/K_m_)), where *L* denotes the radiolabel concentration [105]. The calculated K_i_ was in turn used to determine the Gibbs free energy (ΔG^0^) of the inhibitor–transporter interaction using the equation: ΔG^0^ = -RTln(K_i_), where R represents the gas constant and T symbolizes the absolute temperature in Kelvin [104].

## 5. Conclusions

We have fully characterized the substrate specificity and chemotherapeutic potential of TcoAT1. The presented data adds to the evidence that this transporter is purely a P1-type nucleoside transporter with no role in the uptake and sensitivity of trypanocides in current clinical use. In addition, we have cloned and characterized the functions of a *T. vivax* purine transporter (TvxNT3) for the first time, which is also a P1-type transporter, although with higher permissibility for nucleobases. We concluded that the very similar substrate recognition by the TcoAT1, TvxNT3 and *T. brucei* P1 transporters can be utilized in designing nucleoside drugs for the treatment of nagana. A series of P1-type 7-deaza nucleoside substrates was designed and tested; promising activity was found against the five major pathogenic trypanosomes of veterinary importance. Most of these compounds presented low toxicity to mammalian cells, no cross resistance to existing trypanocides, and by-passed the multidrug carrier P2/AT1. Preliminary observations regarding the mechanism of action of these nucleoside drugs show major impacts on mitosis.

## Figures and Tables

**Table 1:** K_m_ and K_i_ values for nucleosides on the transport of [^3^H]-adenosine by *T. congolense* IL3000 and of *T. b. brucei* P1.

Compound	Tc K_m_ or K_i_ (µM)	ΔG^0^ (kJ/mol)	n	Tbb P1 ^1^ (µM)	ΔG^0^ (kJ/mol)
Adenosine	0.48 ± 0.03	−36.0	3	0.36 ± 0.05	−36.8
Tubercidin	403 ± 120	−19.3	3	78 ± 6.4	−23.4
Cordycepin	12.0 ± 2	−28.0	3	210 ± 48	−21.0
Inosine	0.27 ± 0.02	−37.5	3	0.44 ± 0.10	−36.3
Guanosine	1.06 ± 0.07	−34.1	3	1.8 ± 0.3	−32.7
Uridine	390 ± 9.1	−19.4	3	830 ± 86	−18.7
Thymidine	94.6 ± 11.5	−23.0	3	44 ± 10	−11.4
Cytidine	1176 ± 148	−16.7	3	>250	

IC_50_ values obtained were converted to K_i_ based on the K_m_ of adenosine and determined using a [^3^H]-adenosine concentration of 0.05 µM. All experiments were performed in triplicate. IC_50_ values were based on a minimum of 5 points on the curve, which was extrapolated to zero when necessary but only when inhibition at the highest concentration already exceeded 50%. ΔG^0^, Gibbs free energy of interaction. ^1^ values taken from De Koning and Jarvis [44] for easy side-by-side comparison.

**Table 2:** ENT family genes of *T. vivax*.

Gene ID	Given Name	Number of Amino Acids	Number of TMDs	Closest Related *T. brucei* Transporter	Remarks
**Nucleoside transporters**					
TcIL3000_9_2500	*TcoAT1*/*TcoNT10*	472	10	TbNT10	P1-type
TvY486_0202110	TvxNT1	472	11	TbNT4	P1-type
TvY486_1112030	TvxNT2	470	11	TbNT12	P1-type
TvY486_0043680	TvxNT3	464	10	TbNT6	P1-type
TvY486_0014570	TvxNT4	373	7	TbNT10	P1-type
**Nucleobase transporters**					
TvY486_0011610	(fragment)	284	6	NT8	-----
TvY486_1103750	(fragment)	150	4	NT8	-----
TvY486_1103760	TvxNT5	385	9	NT8	NBT
TvY486_0041960	TvxNT6	437	10	NT8	NBT
TvY486_1103740	TvxNT7	436	10	NT8	NBT

Gene IDs from TriTrypDB. NBT, nucleobase transporter. TMDs called by TMHMM—2.0.

**Table 3:** K_i_ for purines on the transport of 0.05 µM [^3^H]-adenosine by TcoAT1 expressed in SUPKO.

Purine	K_m_ or K_i_ (μM)	ΔG^0^	δ(ΔG^0^)	n
Adenosine	0.42 ± 0.03	−36.4		3
Inosine	0.55 ± 0.09	−35.7	0.68	3
Guanosine	0.80 ± 0.09	−34.8	1.62	3
2′-deoxyadenosine	0.50 ± 0.07	−36.0	0.44	3
5′-deoxyadenosine	0.45 ± 0.08	−36.2	0.18	3
Cordycepin	4.59 ± 0.63	−30.5	5.94	3
2′,3′-dideoxyinosine	85.4 ± 7.1	−23.2	12.51	3
2′,3′-dideoxyadenosine	51.9 ± 2.6	−24.4	11.95	3
1-deazaadenosine	1.03 ± 0.34	−34.2	2.24	4
3-deazaadenosine	138 ± 37	−22.0	14.37	3
Tubercidin	754 ± 54.3	−17.8	18.58	3
Uridine	1467 ± 172	−16.2	20.23	3
Thymidine	434 ± 46.4	−19.2	17.21	4
Cytidine	1577 ± 69.2	−16.0	20.41	3
Adenine	761± 103	−17.8	18.60	5
Hypoxanthine	61.2 ± 3.26	−24.0	11.68	3

EC_50_ values obtained were converted to K_i_ based on the K_m_ of adenosine. All experiments were performed in triplicate. IC_50_ values were based on a minimum of 5 points on the curve, which was extrapolated to zero when necessary but only when inhibition at the highest concentration already exceeded 50%.

**Table 4:** K_i_ for purines on the transport of [^3^H]-adenosine by TvxNT3.

	K_i_				
	AVG	SEM	n	ΔG^0^	δ(ΔG^0^)
Adenosine	1.41	0.35	3	−33.38	
Guanosine	1.37	0.13	3	−33.45	0.07
Inosine	0.18	0.05	3	−38.46	5.08
2′-deoxyadenosine	1.06	0.29	3	−34.1	0.70
5′-deoxyadenosine	1.10	0.12	3	−34.0	0.63
Cordycepin	8.6	1.4	3	−28.91	−4.47
Tubercidin	287	55	3	−20.21	−13.17
Nebularine	4.6	1.1	4	−30.4	−2.94
6-thioinosine	4.03	0.78	4	−30.8	−7.7
3-deazainosine	33.0	17.4	4	−25.6	−12.9
Adenine	2.43	0.14	2	−32.04	−1.34
Hypoxanthine	1.75	0.17	3	−32.85	−7.45
Uridine	720	110	3	−17.93	−15.45
Thymidine	603	51	3	−18.37	−15.01

IC_50_ values obtained were converted to K_i_ based on the K_m_ of adenosine. See legends to Table 1 and Table 3 for further information.

**Table 5:** Effect of the expression of nucleoside transporters on the sensitivity of tubercidin analogs.

Compound	3′	6-Subst	7-Subst	*Lmex*Cas9	SUPKO		SUPKO+TcoAT1		SUPKO+TvxNT3	
				EC_50_ (µM)	EC_50_ (µM)	RF (Cas9)	EC_50_ (µM)	RF (SUPKO)	EC_50_ (µM)	RF (SUPKO)
**Tubercidin**	OH	NH_2_		0.44 ± 0.06	>100	>230	>100	-	>100	-
**Cordycepin**		NH_2_		91.6 ± 12.9	>100	>1.09	>100	-	>100	-
**FH7429_u**		NH_2_	Br	27.3 ± 7.0	>200	>3.6	27.0 ± 5.8	>7.4	42.9 ± 12.0	>2.33
**FH7429_d**		NH_2_		15.1 ± 1.7	>100	>6.63	65.3 ± 7.6	>1.53	>100	-
**FH6367**	OH	NH_2_	CF_3_	26.1 ± 6.4	>100	>3.83	3.79 ± 0.34	>26.4	7.4 ± 1.4	>13.5
**FH3169**	OH	NH_2_	Cl	0.23 ± 0.005	15.4 ± 2.7	67.7	9.41 ± 0.56	1.64	15.9 ± 2.5	0.97
**TH1008**	OH	NH_2_	Pyridin-2-yl	11.7 ± 0.5	>100	>8.5	>100	-	>100	-
**TH1003**	OH	NH_2_	Br	2.43 ± 0.14	>100	>41.2	71.1 ± 5.0	>1.4	>100	-
**FH3167**	OH	NH_2_	F	0.096 ± 0.009	63.4 ± 5.5	660	7.5 ± 0.9	8.48	57.2 ± 6.8	1.1
**CL4510**	OH	OCH_3_		0.39 ± 0.05	2.28 ± 0.21	5.84	2.2 ± 0.03	1.03	2.05 ± 0.04	1.1
**FH8517**		NH_2_	F	3.09 ± 0.04	9.96 ± 2.04	3.22	6.1 ± 0.7	1.64	9.42 ± 1.41	1.06
**FH8505**		NH_2_	ethynyl	92.7 ± 4.3	>100	>1.08	89.9 ± 12.5	>1.1	80.0 ± 7.7	>1.25
**FH8496**		NH_2_	I	51.8 ± 10.3	52.6 ± 0.0	1.01	23.9 ± 5.6	>2.2	40.0 ± 9.2	>1.2
**FH9531**	OH	O-*i*Pr	Cl	>100	>100	-	>100	-	>100	-
**FH10659**		NH_2_	-vinyl	>100	>100	-	>100	-	>100	-
**Pentamidine**				1.43 ± 0.05	1.83 ± 0.29	1.28	1.73 ± 0.20	1.1	2.31 ± 0.18	0.77

EC_50_ values were obtained using the resazurin-based assay in 96-well plates, with doubling dilutions over 1 or 2 rows (11 or 23 dilutions) as necessary to obtain a full EC_50_. Data listed are the average of 3 or more experiments and SEM. When inhibition was less than 50% at 100 µM the value is listed as >100. RF is the Resistance Factor relative to the cell line given in brackets, e.g., RF(cas9) = EC_50_(SUPKO)/EC_50_(Cas9).

**Table 6:** K_m_ or K_i_, ΔG^0^ and δ(ΔG^0^) for toxic nucleosides on the transport of [^3^H]-adenosine by TcoAT1 expressed in *L. mexicana* SUPKO and by *T. congolense* IL3000 bloodstream forms.

	TcoAT1 in *L. mexicana* SUPKO			*T. congolense* IL300		
	K_m_ or K_i_ (μM)	ΔG^0^ (kJ/mol)	n	K_m_ or K_i_ (μM)	ΔG^0^ (kJ/mol)	n
Adenosine	0.42 ± 0.03	−36.4	3	0.48 ± 0.03	−36.0	3
Tubercidin	693 ± 25	−17.8	3	403 ± 120	−19.4	3
Cordycepin	4.6 ± 0.6	−30.5	3	12.0 ± 2.0	−28.1	3
FH7429_u	259 ± 11	−20.5	3			
FH7429_d	810 ± 60	−18.0	3	319 ± 93	−19.9	3
FH8496	135 ± 11	−20.6	3	46.2 ± 1.7	−24.7	3
FH8470	60.6 ± 10.7	−24.1	3	17.5 ± 3.4	−27.1	4
FH8517	229 ± 15	−20.8	3	98.1 ± 2.3	−22.9	3
FH10659	59.3 ± 4.3	−17.7	4	28.5 ± 8.1	−25.9	6
TH1012	146 ± 58	−21.9	3			
CL4510	226 ± 26	−20.8	3			

IC_50_ values obtained were converted to K_i_ based on the K_m_ of adenosine. See Legend to Table 1 and Table 3.

**Table 7:** K_i_ for toxic nucleosides on the transport of [^3^H]-adenosine by TvxNT3 expressed in SUPKO.

	K_m_ or K_i_ (µM)	ΔG^0^ (kJ/mol)	n
Adenosine	1.41 ± 0.35	−33.4	3
Tubercidin	287 ± 55	−20.2	3
Cordycepin	8.57 ± 1.45	−28.9	3
FH 7429_u	169 ± 4	−21.5	3
FH 7429_d	388 ± 13	−19.5	3
CL4510	16.1 ± 2.8	−27.4	3
CL5565	32.0 ± 9.2	−25.7	3
FH15963	225 ± 42	−20.8	3
FH15967	109 ± 30	−22.6	3

IC_50_ values obtained were converted to K_i_ based on the K_m_ of adenosine. See Legend to Table 1 and Table 3.

**Table 8:** Effect of tubercidin analogs with substitutions on C7 on *Trypanosoma* species and strains in vitro.

					*T. congolense* IL3000	*T. congolense* 6C3		*T. vivax* **	*T.b.b*. B48	*T. evansi*	*T. equiperdum*
Compound	C7	2′-OH	3′-OH	6-Position	EC_50_ (µM)	EC_50_ (µM)	RF ^†^	EC_50_ (µM)	EC_50_ (µM)	EC_50_ (µM)	EC_50_ (µM)
Tubercidin	None	+	+	NH_2_	3.16 ± 0.50	1.83 ± 0.39	0.58		4.30 ± 1.30		
Cordycepin	None	+	−	NH_2_	2.46 ± 0.42	2.16 ± 0.44	0.88	0.12 ± 0.02			
FH7429_d *	None	+	−	NH_2_	0.021 ± 0.001 *	0.019 ± 0.002 *	0.91	3.48 ± 0.09	0.25 ± 0.02	0.017 ± 0.002	0.0026 ± 0.0006
FH10677	None	−	+	NH_2_	43.3 ± 14.5	10.9 ± 2.3	0.25	>64.0	38.0 ± 2.3	>100	17.4 ± 2.3
FH7429_u	Br	+	−	NH_2_	0.00069 ± 0.00007	0.00049 ± 0.00009	0.71	0.018 ± 0.003	0.0048 ± 0.0005	0.00090 ± 0.00022	0.00051 ± 0.00007
FH8496	I	+	−	NH_2_	0.0054 ± 0.0010	0.0030 ± 0.0002	0.56	0.056 ± 0.001	0.024 ± 0.002	0.0037 ± 0.0012	0.0015 ± 0.0005
FH8470 *	Cl	+	−	NH_2_	0.0012 ± 0.0002	0.00066 ± 0.00003	0.53 ^a^	0.096 ± 0.082	0.0068 ± 0.0009	0.0018 ± 0.0002	0.00057 ± 0.00008
FH8517	F	+	−	NH_2_	0.0052 ± 0.0003	0.0031 ± 0.0003	0.60	0.50 ± 0.01	0.013 ± 0.002	0.0040 ± 0.0006	0.00052 ± 0.00011
FH10679	Br	−	+	NH_2_	0.51 ± 0.09	0.31 ± 0.07	0.60	4.30 ± 0.90	1.04 ± 0.19	1.74 ± 0.53	0.067 ± 0.013
TH1003	Br	+	+	NH_2_	0.79 ± 0.14	0.52 ± 0.031	0.66	0.74 ± 0.04	1.40 ± 0.09	0.48 ± 0.06	0.085 ± 0.026
FH3169	Cl	+	+	NH_2_	0.86 ± 0.12	0.75 ± 0.14	0.87	0.053 ± 0.007	1.84 ± 0.33	0.62 ± 0.06	0.12 ± 0.018
FH3167	F	+	+	NH_2_	0.45 ± 0.62	0.48 ± 0.10	1.06	0.038 ± 0.006	0.16 ± 0.03	0.18 ± 0.023	0.032 ± 0.007
FH6367	CF3	+	+	NH_2_	1.80 ± 0.44	0.89 ± 0.17	0.50	0.82 ± 0.09	0.90 ± 0.13	0.68 ± 0.11	0.19 ± 0.03
FH9531	Cl	+	+	O-*i*Pr	4.04 ± 0.36 *	3.23 ± 0.36 *	0.80	0.12 ± 0.01			
FH8505	ethynyl	+	−	NH_2_	0.017 ± 0.002	0.0080 ± 0.002	0.46 ^a^	0.16 ± 0.01	0.031 ± 0.004	0.0078 ± 0.0020	0.0026 ± 0.0008
FH10659	vinyl	+	−	NH_2_	0.056 ± 0.004	0.040 ± 0.003	0.70	1.57 ± 0.43		0.038 ± 0.0015	0.011 ± 0.001
TH1008 *	Pyridin-2-yl	+	+	NH_2_	0.17 ± 0.05	0.056 ± 0.013	0.33	12.9 ± 4.4	0.39 ± 0.03	0.040 ± 0.005	0.061 ± 0.005
Diminazene					0.23 ± 0.04	1.32 ± 0.43	5.7 ^a^		0.34 ± 0.13		
Pentamidine					0.55 ± 0.11	0.44 ± 0.15	0.79		0.34 ± 0.06		

All EC_50_ values were obtained by Alamar Blue (resazurin) assays and are shown as averages in nM (±SEM) of at least 3 independent determinations. EC_50_ values were obtained using the resazurin-based assay in 96-well plates, with doubling dilutions over 1 or 2 rows (11 or 23 dilutions) as necessary to obtain a full EC_50_. When inhibition was less than 50% at 100 µM the value is listed as >100. 6C3 = diminazene resistant; B48 = multidrug-resistant *T. brucei* strain. ^†^ RF = resistance factor, in comparison to wild-type or parental strain, being EC_50_ (test strain)/EC_50_(control strain). ^a^ *p* ≤ 0.05 by unpaired Student’s *t-*test. * EC_50_ obtained from Hulpia et al. [38]. ** EC_50_ obtained from Mabille et al. [69], added for comparison only.

**Table 9:** Toxicity of tubercidin analogs with substitutions on C7 against human embryonic kidney (HEK) cells.

	HEK EC_50_ (µM)	SI ^†^ *T. evansi*	SI ^†^ *T. equiperdum*	SI ^†^ *T. congolense*
**FH7429_u**	2.18 ± 0.41	2430	4271	3158
**FH7429_d**	>200	>11,800	>76,000	9700
**FH6367**	0.49 ± 0.01	0.7	2.6	0.3
**FH3169**	4.55 ± 0.19	7.4	37	5.3
**TH1008**	18.5 ± 1.5	466	303	110
**TH1003**	2.37 ± 0.12	4.9	28	3.0
**FH8470**	12.0 ± 0.32	6830	21,100	9800
**FH3167**	0.49 ± 0.05	2.7	15	1.1
**FH8517**	1.71 ± 0.11	431	3310	330
**FH8505**	1.17 ± 0.06	151	446	68
**FH8496**	4.91 ± 0.03	1340	3260	902
**FH10679**	>200	>115	>3000	>390
**FH10677**	>200		>12	>4.6

^†^ SI = selectivity index of parasite species in comparison to HEK cells, being EC_50_ (HEK cells)/EC_50_ (parasite species). The EC_50_s were determined using resazurin-based assays in 96 well plates, using doubling dilutions from 100 µM across one row (11-dilutions). Data shown are the average and SEM of at least 3 independent determinations.

**Table 10:** Kinetic parameters of nucleoside transporters of *T. brucei brucei, T. congolense* and *T. vivax*.

Transporter	Substrate								
	ADE	ADO	GUA	GUO	HYP	INO	CTD	TMD	URD
TbP2/AT1	0.38 ^a^; 0.30 ^c^	**0.59** ^a^; **0.92** ^c^		NE ^3,c^	>500 ^c^	NE ^4,c^	NE ^3,c^	NE ^2,c^	NE ^2,c^
TbP1/NT2	NE ^3,c^	**0.15** ^a^; **0.26** ^b^; **0.38** ^c^		1.8 ^c^	NE ^1,c^	0.44 ^c^	NE ^3,c^	44 ^c^	830 ^c^
TbNT5 ^d^		**2**	**2.2**		**49.4**				
TbNT6 ^d^		**1.4**	**4.3**						
TbNT7 ^d^		**0.3**	**1.8**						
TbNT9 ^e^	148	**0.068**		6.2	320	**2.75**		510	235
TbNT10 ^e^		**0.41**				**0.53**			
TbNT11 ^f^	**2.7**; 8	266	651		**141**				
TbNT8/H4 ^g^	**2.6**	860	2.6	4.7	**0.55**	20			95
TcoAT1	786	**0.42**		0.80	61.2	0.55	1630	49.6	147
TvxNT3	2.43	**1.41**		1.37	1.75	0.086	>100	603	720

Values in bold represent K_m_ values; all others are K_i_ values. ADE, adenine; ADO, adenosine; GUA, guanine; GUO, guanosine; HYP, hypoxanthine; INO, inosine; CTD, cytidine; TMD, thymidine; URD, uridine; NE, no effect at ^1^ 1 mM, ^2^ 500 μM, ^3^ 250 μM, ^4^ 100 μM. ^a^ Carter and Fairlamb [41]; ^b^ De Koning et al. [34]; ^c^ De Koning and Jarvis [44]; ^d^ Sanchez et al. [39]; ^e^ Al-Salabi et al. [40]); ^f^ Ortiz et al. [96]; ^g^ Burchmore et al. [70]. Data for *T. congolense* (Tco) and *T. vivax* (Tvx) transporters were from this study. NE, no effect at the stated concentration.

## Data Availability

All relevant data are included in the manuscript and Supplemental Materials.

## Supplementary Materials

The following supporting information can be downloaded at: https://www.mdpi.com/article/10.3390/ijms24043144/s1.

## References

[1] Giordani F., Morrison L.J., Rowan T.G., De Koning H.P., Barrett M.P. (2016). The animal trypanosomiases and their chemotherapy: A review. Parasitology.

[2] Franco J.R., Cecchi G., Priotto G., Paone M., Diarra A., Grout L., Simarro P.P., Zhao W., Argaw D. (2020). Monitoring the elimination of human African trypanosomiasis at continental and country level: Update to 2018. PLoS Negl. Trop. Dis..

[3] Autheman D., Crosnier C., Clare S., Goulding D.A., Brandt C., Harcourt K., Tolley C., Galaway F., Khushu M., Ong H. (2021). An invariant *Trypanosoma vivax* vaccine antigen induces protective immunity. Nature.

[4] La Greca F., Magez S. (2011). Vaccination against trypanosomiasis: Can it be done or is the trypanosome truly the ultimate immune destroyer and escape artist?. Hum. Vaccin..

[5] Magez S., Li Z., Nguyen H.T.T., Pinto Torres J.E., Van Wielendaele P., Radwanska M., Began J., Zoll S., Sterckx Y.G. (2021). The history of anti-trypanosome vaccine development shows that highly immunogenic and exposed pathogen-derived antigens are not necessarily good target candidates: Enolase and ISG75 as examples. Pathogens.

[6] Delespaux V., De Koning H.P. (2007). Drugs and drug resistance in African trypanosomiasis. Drug Resist. Updat..

[7] De Koning H.P. (2020). The drugs of sleeping sickness: Their mechanisms of action and resistance, and a brief history. Trop. Med. Infect. Dis..

[8] Desquesnes M., Dargantes A., Lai D.H., Lun Z.R., Holzmuller P., Jittapalapong S. (2013). *Trypanosoma evansi* and surra: A review and perspectives on transmission, epidemiology and control, impact, and zoonotic aspects. Biomed. Res. Int..

[9] Osório A.L., Madruga C.R., Desquesnes M., Soares C.O., Ribeiro L.R., Costa S.C. (2008). *Trypanosoma* (Duttonella) *vivax*: Its biology, epidemiology, pathogenesis, and introduction in the New World–a review. Mem. Inst. Oswaldo Cruz.

[10] Gizaw Y., Megersa M., Fayera T. (2017). Dourine: A neglected disease of equids. Trop. Anim. Health Prod..

[11] Anene B.M., Onah D.N., Nawa Y. (2001). Drug resistance in pathogenic African trypanosomiasis: What hopes for the future?. Vet. Parasitol..

[12] Wilkinson S.R., Kelley J.M. (2009). Trypanocidal drugs: Mechanisms, resistance and new targets. Expert Rev. Mol. Med..

[13] Morrison L.J., Vezza L., Rowan T., Hope J.C. (2016). Animal African trypanosomiasis: Time to increase focus on clinically relevant parasite and host species. Trends Parasitol..

[14] Zweygarth E., Kaminsky R. (1990). Evaluation of an arsenical compound (RM 110, mel Cy, Cymelarsan) against susceptible and drug-resistant *Trypanosoma brucei brucei* and *T. b. evansi*. Trop. Med. Parasitol..

[15] Kinabo L.D.B. (1993). Pharmacology of existing drugs for animal trypanosomiasis. Acta Trop..

[16] Geerts S., Holmes P.H., Eisler M.C., Diall O. (2001). African bovine trypanosomiasis: The problem of drug resistance. Trends Parasitol..

[17] Munday J.C., Settimo L., De Koning H.P. (2015). Transport proteins determine drug sensitivity and resistance in a protozoan parasite, *Trypanosoma brucei*. Front. Pharmacol..

[18] Ungogo M.A., Campagnaro G.D., Alghamdi A.H., Natto M.J., De Koning H.P. (2022). Differences in transporters rather than drug targets are the principal determinants of the different innate sensitivities of *Trypanosoma congolense* and *Trypanozoon* subgenus trypanosomes to diamidines and melaminophenyl arsenicals. Int. J. Mol. Sci..

[19] Vincent I.M., Creek D., Watson D.G., Kamleh M.A., Woods D.J., Wong P.E., Burchmore R.J., Barrett M.P. (2010). A molecular mechanism for eflornithine resistance in African trypanosomes. PLoS Pathog..

[20] Stewart M.L., Burchmore R.J.S., Clucas C., Hertz-Fowler C., Brook K., Tait A., McLeod A., Turner C.M.R., De Koning H.P., Wong P.E. (2010). Multiple genetic mechanisms lead to the loss of functional TbAT1 expression in drug resistant trypanosomes. Eukaryot. Cell.

[21] Baker N., De Koning H.P., Mäser P., Horn D. (2013). Drug resistance in African trypanosomiasis: The melarsoprol and pentamidine story. Trends Parasitol..

[22] Alghamdi A.H., Munday J.C., Campagnaro G.D., Gurvic D., Svensson F., Okpara C.E., Kumar A., Quintana J., Martin Abril M.E., Milić P. (2020). Positively selected modifications in the pore of TbAQP2 allow pentamidine to enter *Trypanosoma brucei*. eLife.

[23] Kell D.B. (2021). The transporter-mediated cellular uptake and efflux of pharmaceutical drugs and biotechnology products: How and why phospholipid bilayer transport Is negligible in real biomembranes. Molecules.

[24] Hassan H.F., Coombs G.H. (1988). Purine and pyrimidine metabolism in parasitic protozoa. FEMS Microbiol. Rev..

[25] De Koning H.P., Bridges D.J., Burchmore R. (2005). Purine and pyrimidine transport in protozoa: From biology to therapy. FEMS Microbiol. Rev..

[26] Campagnaro G.D., De Koning H.P. (2020). Purine and pyrimidine transporters of pathogenic protozoa-conduits for therapeutic agents. Med. Res. Rev..

[27] Campagnaro G.D., de Freitas Nascimento J., Girard R.B.M., Silber A.M., De Koning H.P. (2018). Cloning and characterisation of the Equilibrative Nucleoside Transporter family of *Trypanosoma cruzi*: Ultra-high affinity and selectivity to survive in the intracellular niche. Biochim. Biophys. Acta Gen. Subj..

[28] Campagnaro G.D., Elati H.A.A., Balaska S., Martin Abril M.E., Natto M.J., Hulpia F., Lee K., Sheiner L., Van Calenbergh S., De Koning H.P. (2022). A *Toxoplasma gondii* oxopurine transporter binds nucleobases and nucleosides using different binding modes. Int. J. Mol. Sci..

[29] Aldfer M.M., AlSiari T.A., Elati H.A.A., Natto M.J., Alfayez I.A., Campagnaro G.D., Sani B., Burchmore R.J.S., Diallinas G., De Koning H.P. (2022). Nucleoside transport and nucleobase uptake null mutants in *Leishmania mexicana* for the routine expression and characterisation of purine and pyrimidine transporters. Int. J. Mol. Sci..

[30] Aldfer M.M., Alfayez I.A., Campagnaro G.D., Gayen N., Elmahallawy E.K., Murillo A.M., Marsiccobetre S., Van Calenbergh S., Silber A.M., De Koning H.P. (2022). TcrNT2 is a conduit for the uptake of 5-F-2’deoxyuridine and tubercidin analogues in *Trypanosoma cruzi*. Molecules.

[31] Campagnaro G.D., Alzahrani K.J.H., Munday J.C., De Koning H.P. (2018). *Trypanosoma brucei* bloodstream forms express highly specific and separate transporters for adenine and hypoxanthine; evidence for a new protozoan purine transporter family?. Mol. Biochem. Parasitol..

[32] De Koning H.P. (2007). Pyrimidine transporters of protozoa–A class apart?. Trends Parasitol..

[33] De Koning H.P., Jarvis S.M. (1997). Hypoxanthine uptake through a purine-selective nucleobase transporter in *Trypanosoma brucei brucei* procyclics is driven by protonmotive force. Eur. J. Biochem..

[34] De Koning H.P., Watson C.J., Jarvis S.M. (1998). Characterisation of a nucleoside/proton symporter in procyclic *Trypanosoma brucei brucei*. J. Biol. Chem..

[35] Stein A., Vaseduvan G., Carter N.S., Ullman B., Landfear S.M., Kavanaugh M.P. (2003). Equilibrative nucleoside transporter family members from *Leishmania donovani* are electrogenic proton symporters. J. Biol. Chem..

[36] Young J.D., Yao S.Y., Baldwin J.M., Cass C.E., Baldwin S.A. (2013). The human concentrative and equilibrative nucleoside transporter families, SLC28 and SLC29. Mol. Aspects Med..

[37] Boswell-Casteel R.C., Hays F.A. (2017). Equilibrative nucleoside transporters–a review. Nucleosides Nucleotides Nucleic Acids.

[38] Hulpia F., Mabille D., Campagnaro G.D., Schumann G., Maes L., Roditi I., Hofer A., De Koning H.P., Galjon G., Van Calenbergh S. (2019). Combining tubercidin and cordycepin scaffolds results in highly active candidates to treat late-stage sleeping sickness. Nat. Commun..

[39] Sanchez M.A., Tryon R., Green J., Boor I., Landfear S.M. (2002). Six related nucleoside/nucleobase transporters from *Trypanosoma brucei* exhibit distinct biochemical functions. J. Biol. Chem..

[40] Al-Salabi M.I., Wallace L.J.M., Lüscher A., Mäser P., Candlish D., Rodenko B., Gould M.K., Jabeen I., Ajith S.N., De Koning H.P. (2007). Molecular interactions underlying the unusually high adenosine affinity of a novel *Trypanosoma brucei* nucleoside transporter. Mol. Pharmacol..

[41] Carter N.S., Fairlamb A.H. (1993). Arsenical-resistant trypanosomes lack an unusual adenosine transporter. Nature.

[42] Mäser P., Sütterlin C., Kralli A., Kaminsky R. (1999). A nucleoside transporter from *Trypanosoma brucei* involved in drug resistance. Science.

[43] Carter N.S., Barrett M.P., De Koning H.P. (1999). A drug resistance determinant from *Trypanosoma brucei*. Trends Microbiol..

[44] De Koning H.P., Jarvis S.M. (1999). Adenosine transporters in bloodstream forms of T. b. brucei: Substrate recognition motifs and affinity for trypanocidal drugs. Mol. Pharmacol..

[45] Munday J.C., Tagoe D.N.A., Eze A.A., Krezdorn J.A., Rojas López K.E., Alkhaldi A.A.M., McDonald F., Still J., Alzahrani K.J., Settimo L. (2015). Functional analysis of drug resistance-associated mutations in the *Trypanosoma brucei* adenosine transporter 1 (TbAT1) and the proposal of a structural model for the protein. Mol. Microbiol..

[46] Geiser F., Lüscher A., De Koning H.P., Seebeck T., Mäser P. (2005). Molecular pharmacology of adenosine transport in *Trypanosoma brucei*: P1/P2 revisited. Mol. Pharmacol..

[47] Vodnala S.K., Lundbäck T., Yeheskieli E., Sjöberg B., Gustavsson A.L., Svensson R., Olivera G.C., Eze A.A., De Koning H.P., Hammarström L.G.J. (2013). Structure-activity relationships of synthetic cordycepin analogues as experimental therapeutics for African trypanosomiasis. J. Med. Chem..

[48] Hulpia F., Campagnaro G.D., Scortichini M., Van Hecke K., Maes L., De Koning H.P., Caljon G., Van Calenbergh S. (2019). Revisiting tubercidin against kinetoplastid parasites: Aromatic substitutions at position 7 improve activity and reduce toxicity. Eur. J. Med. Chem..

[49] Carter N.S., Berger B.J., Fairlamb A.H. (1995). Uptake of diamidine drugs by the P2 nucleoside transporter in melarsen-sensitive and -resistant *Trypanosoma brucei brucei*. J. Biol. Chem..

[50] De Koning H.P., MacLeod A., Barrett M.P., Cover B., Jarvis S.M. (2000). Further evidence for a link between melarsoprol resistance and P2 transporter function in African trypanosomes. Mol. Biochem. Parasitol..

[51] Witola W.H., Inoue N., Ohashi K., Onuma M. (2004). RNA-interference silencing of the adenosine transporter-1 gene in *Trypanosoma evansi* confers resistance to diminazene aceturate. Exp. Parasitol..

[52] Munday J.C., Rojas López K.E., Eze A.A., Delespaux V., Van Den Abbeele J., Rowan T., Barrett M.P., Morrison L.J., De Koning H.P. (2013). Functional expression of TcoAT1 reveals it to be a P1-type nucleoside transporter with no capacity for diminazene uptake. Int J. Parasitol. Drugs Drug Resist..

[53] Jackson A.P., Allison H.C., Barry J.D., Field M.C., Hertz-Fowler C., Berriman M. (2013). A cell-surface phylome for African trypanosomes. PLoS Negl. Trop. Dis..

[54] Delespaux V., Chitanga S., Geysen D., Goethals A., Van den Bossche P., Geerts S. (2006). SSCP analysis of the P2 purine transporter TcoAT1 gene of *Trypanosoma congolense* leads to a simple PCR-RFLP test allowing the rapid identification of diminazene resistant stocks. Acta Trop..

[55] Vitouley H.S., Mungube E.O., Allegye-Cudjoe E., Diall O., Bocoum Z., Diarra B., Randolph T.F., Bauer B., Clausen P.H., Geysen D. (2011). Improved PCR-RFLP for the detection of diminazene resistance in Trypanosoma congolense under field conditions using filter papers for sample storage. PLoS Negl. Trop. Dis..

[56] Mamoudou A., Delespaux V., Chepnda V., Hachimou Z., Andrikaye J.P., Zoli A., Geerts S. (2008). Assessment of the occurrence of trypanocidal drug resistance in trypanosomes of naturally infected cattle in the Adamaoua region of Cameroon using the standard mouse test and molecular tools. Acta Trop..

[57] Mewamba E.M., Farikou O., Kamga R.M.N., Magang M.E.K., Tume C., Tiofack A.A.Z., Ravel S., Simo G. (2020). Molecular identification of diminazene aceturate-resistant strains of *Trypanosoma congolense* in naturally infected domestic animals of Yoko in the centre region of Cameroon. Vet. Parasitol. Reg. Stud. Rep..

[58] Simo G., Magang E.M.K., Mewamba E.M., Farikou O., Kamga R.M.N., Tume C., Solano P., Ravel S. (2020). Molecular identification of diminazene aceturate resistant trypanosomes in tsetse flies from Yoko in the Centre region of Cameroon and its epidemiological implications. Parasite Epidemiol. Control.

[59] Bridges D., Gould M.K., Nerima B., Mäser P., Burchmore R.J.S., De Koning H.P. (2007). Loss of the High Affinity Pentamidine Transporter is responsible for high levels of cross-resistance between arsenical and diamidine drugs in African trypanosomes. Mol. Pharmacol..

[60] Carruthers L.V., Munday J.C., Ebiloma G.U., Steketee P., Jayaraman S., Campagnaro G.D., Ungogo M.A., Donnachie A., Lemgruber L., Rowan T.G. (2021). Diminazene resistance in *Trypanosoma congolense* is not caused by reduced transport capacity but associated with reduced mitochondrial membrane potential. Mol. Microbiol..

[61] Alzahrani K.J.H., Ali J.A.M., Eze A.A., Looi W.L., Tagoe D.N.A., Creek D.J., Barrett M.P., De Koning H.P. (2017). Functional and genetic evidence that nucleoside transport is highly conserved in *Leishmania* species: Implications for pyrimidine-based chemotherapy. Int. J. Parasitol. Drugs Drug Resist..

[62] Vasudevan G., Carter N.S., Drew M.E., Beverley S.M., Sanchez M.A., Seyfang A., Ullman B., Landfear S.M. (1998). Cloning of Leishmania nucleoside transporter genes by rescue of a transport-deficient mutant. Proc. Natl. Acad. Sci. USA.

[63] Carter N.S., Drew M.E., Sanchez M., Vasudevan G., Landfear S.M., Ullman B. (2000). Cloning of a novel inosine-guanosine transporter gene from *Leishmania donovani* by functional rescue of a transport-deficient mutant. J. Biol. Chem..

[64] Beneke T., Madden R., Makin L., Valli J., Sunter J., Gluenz E. (2017). A CRISPR Cas9 high-throughput genome editing toolkit for kinetoplastids. R. Soc. Open Sci..

[65] Hulpia F., Campagnaro G.D., Alzahrani K.J., Alfayez I.A., Ungogo M.A., Mabille D., Maes L., De Koning H.P., Caljon G., Van Calenbergh S. (2020). Structure-activity relationship exploration of 3′-deoxy-7-deazapurine nucleoside analogues as anti-*Trypanosoma brucei* agents. ACS Infect. Dis..

[66] Hulpia F., Bouton J., Campagnaro G.D., Alfayez I.A., Mabille D., Maes L., De Koning H.P., Caljon G., Van Calenbergh S. (2020). C6-O-alkylated 7-deazainosine nucleoside analogues: Discovery of potent and selective anti-sleeping sickness agents. Eur. J. Med. Chem..

[67] De Almeida Fiuza L.F., Batista D.G.J., Girão R.D., Hulpia F., Finamore P., Aldfer M.M., Elmahallawy E.K., De Koning H.P., Moreira O.C., Van Calenbergh S. (2022). Phenotypic evaluation of nucleoside analogues against *Trypanosoma cruzi* infection: In vitro and in vivo approaches. Molecules.

[68] Natto M.J., Hulpia F., Kalkman E.R., Baillie S., Alhejeli A., Miyamoto Y., Eckmann L., Van Calenbergh S., De Koning H.P. (2021). Deazapurine nucleoside analogues for the treatment of *Trichomonas vaginalis*. ACS Infect. Dis..

[69] Mabille D., Ilbeigi K., Hendrickx S., Ungogo M.A., Hulpia F., Lin C., Maes L., De Koning H.P., Van Calenbergh S., Caljon G. (2022). Nucleoside analogues for the treatment of animal African trypanosomiasis. Int. J. Parasitol. Drugs Drug Resist..

[70] Burchmore R., Wallace L.J.M., Candlish D., Al-Salabi M.I., Beal P., Barrett M.P., Baldwin S.A., De Koning H.P. (2003). Cloning, heterologous expression, and *in situ* characterization of the first high affinity nucleobase transporter from a protozoan. J. Biol. Chem..

[71] Henriques C., Sanchez M.A., Tryon R., Landfear S.M. (2003). Molecular and functional characterization of the first nucleobase transporter gene from African trypanosomes. Mol. Biochem. Parasitol..

[72] Tetaud E., Lecuix I., Sheldrake T., Baltz T., Fairlamb A.H. (2002). A new expression vector for *Crithidia fasciculata* and *Leishmania*. Mol. Biochem. Parasitol..

[73] Gudin S., Quashie N.B., Candlish D., Al-Salabi M.I., Jarvis S.M., Ranford-Cartwright L.C., De Koning H.P. (2006). *Trypanosoma brucei*: A survey of pyrimidine transport activities. Exp. Parasitol..

[74] Ali J.A.M., Creek D.J., Burgess K., Allison H.C., Field M.C., Mäser P., De Koning H.P. (2013). Pyrimidine salvage in Trypanosoma brucei bloodstream forms and the trypanocidal action of halogenated pyrimidines. Mol. Pharmacol..

[75] Munday J.C., Eze A.A., Baker N., Glover L., Clucas C., Aguinaga Andrés D., Natto M.J., Teka I.A., McDonald J., Lee R.S. (2014). *Trypanosoma brucei* Aquaglyceroporin 2 is a high affinity transporter for pentamidine and melaminophenyl arsenic drugs and is the main genetic determinant of resistance to these drugs. J. Antimicrob. Chemother..

[76] Matovu E., Stewart M., Geiser F., Brun R., Mäser P., Wallace L.J.M., Burchmore R.J., Enyaru J.C.K., Barrett M.P., Kaminsky R. (2003). Mechanisms of Arsenical and Diamidine Uptake and Resistance in *Trypanosoma brucei*. Eukaryot. Cell.

[77] Graf F.E., Ludin P., Wenzler T., Kaiser M., Brun R., Pati Pyana P., Büscher P., De Koning H.P., Horn D., Mäser P. (2013). Aquaporin 2 mutations in *Trypanosoma brucei gambiense* field isolates correlate with decreased susceptibility to pentamidine and melarsoprol. PLoS Negl. Trop. Dis..

[78] Hammarton T.C. (2007). Cell cycle regulation in *Trypanosoma brucei*. Mol. Biochem. Parasitol..

[79] Thomas J.A., Baker N., Hutchinson S., Dominicus C., Trenaman A., Glover L., Alsford S., Horn D. (2018). Insights into antitrypanosomal drug mode-of-action from cytology-based profiling. PLoS Negl. Trop. Dis..

[80] Ibrahim H.M., Al-Salabi M.I., El Sabbagh N., Quashie N.B., Alkhaldi A.A., Escale R., Smith T.K., Vial H.J., De Koning H.P. (2011). Symmetrical choline-derived dications display strong anti-kinetoplastid activity. J. Antimicrob. Chemother..

[81] Giordani F., Khalaf A.I., Gillingwater K., Munday J.C., De Koning H.P., Suckling C.J., Barrett M.P., Scott F.J. (2019). Novel Minor Groove Binders cure animal African trypanosomiasis in an in vivo mouse model. J. Med. Chem..

[82] De Koning H.P., Gould M.K., Sterk G.J., Tenor H., Kunz S., Luginbuehl E., Seebeck T. (2012). Pharmacological validation of *Trypanosoma brucei* phosphodiesterases as novel drug targets. J. Infect. Dis..

[83] Nvau J.B., Alenezi S., Ungogo M.A., Alfayez I.A.M., Natto M.J., Igoli J.O., Gray A.I., Ferro V.A., Watson D.G., De Koning H.P. (2020). Antiparasitic and cytotoxic activity of Bokkosin, a novel diterpene substituted chromanyl benzoquinone from *Calliandra portoricensis*. Front. Chem..

[84] Spector T., Jones T.E., LaFon S.W., Nelson D.J., Berens R.L., Marr J.J. (1984). Monophosphates of formycin B and allopurinol riboside: Interactions with leishmanial and mammalian succino-AMP synthetase and GMP reductase. Biochem. Pharmacol..

[85] Steketee P.C., Dickie E.A., Iremonger J., Crouch K., Paxton E., Jayaraman S., Alfituri O.A., Awuah-Mensah G., Ritchie R., Schnaufer A. (2021). Divergent metabolism between *Trypanosoma congolense* and *Trypanosoma brucei* results in differential sensitivity to metabolic inhibition. PloS Path..

[86] Coustou V., Guegan F., Plazolles N., Baltz T. (2010). Complete in vitro life cycle of *Trypanosoma congolense*: Development of genetic tools. PLoS Negl. Trop. Dis..

[87] Awuah-Mensah G., McDonald J., Steketee P.C., Autheman D., Whipple S., D’Archivio S., Brandt C., Clare S., Harcourt K., Wright G.J. (2021). Reliable, scalable functional genetics in bloodstream-form *Trypanosoma congolense* in vitro and in vivo. PLoS Path..

[88] Chamond N., Cosson A., Blom-Potar M.C., Jouvion G., d’Archivio S., Medina M., Droin-Bergère S., Huerre M., Goyard S., Minoprio P. (2010). *Trypanosoma vivax* infections: Pushing ahead with mouse models for the study of Nagana. I. Parasitological, hematological and pathological parameters. PLoS Negl. Trop. Dis..

[89] d’Archivio S., Medina M., Cosson A., Chamond N., Rotureau B., Minoprio P., Goyard S. (2011). Genetic engineering of *Trypanosoma* (Dutonella) *vivax* and in vitro differentiation under axenic conditions. PLoS Negl. Trop. Dis..

[90] D’Archivio S., Cosson A., Medina M., Lang T., Minoprio P., Goyard S. (2013). Non-invasive in vivo study of the *Trypanosoma vivax* infectious process consolidates the brain commitment in late infections. PLoS Negl. Trop. Dis..

[91] Sanchez M.A., Ullman B., Landfear S.M., Carter N.S. (1999). Cloning and functional expression of a gene encoding a P1 type nucleoside transporter from *Trypanosoma brucei*. J. Biol. Chem..

[92] De Koning H.P. (2001). Transporters in African trypanosomes: Role in drug action and resistance. Int. J. Parasitol..

[93] Lüscher A., De Koning H.P., Mäser P. (2007). Chemotherapeutic strategies against *Trypanosoma brucei*: Drug targets vs. drug targeting. Curr. Pharm. Des..

[94] Ogbunude P.O.J., Ikediobi C.O., Ukoha A.I. (1985). Adenosine cycle in African trypanosomes. Ann. Trop. Med. Parasitol..

[95] James D.M., Born G.V.R. (1980). Uptake of purine bases and nucleosides in African trypanosomes. Parasitology.

[96] Ortiz D., Sanchez M.A., Quecke P., Landfear S.M. (2009). Two novel nucleobase/pentamidine transporters from *Trypanosoma brucei*. Mol. Biochem. Parasitol..

[97] Lin C., Ferreira de Almeida Fiuza L., Cardoso Santos C., Ferreira Nunes D., Cruz Moreira O., Bouton J., Karalic I., Maes L., Caljon G., Hulpia F. (2021). 6-Methyl-7-aryl-7-deazapurine nucleosides as anti-*Trypanosoma cruzi* agents: Structure-activity relationship and in vivo efficacy. ChemMedChem.

[98] Lin C., Hulpia F., Karalic I., De Schepper L., Maes L., Caljon G., Van Calenbergh S. (2021). 6-Methyl-7-deazapurine nucleoside analogues as broad-spectrum antikinetoplastid agents. Int. J. Parasitol. Drugs Drug Resist..

[99] Richards S., Morrison L.J., Torr S.J., Barrett M.P., Manangwa O., Mramba F., Auty H. (2021). Pharma to farmer: Field challenges of optimizing trypanocide use in African animal trypanosomiasis. Trends Parasitol..

[100] Fidalgo L.M., Gille L. (2011). Mitochondria and trypanosomatids: Targets and drugs. Pharm. Res..

[101] Eze A.A., Gould M.K., Munday J.C., Tagoe D.N.A., Stelmanis V., Schnaufer A., De Koning H.P. (2016). Loss of mitochondrial membrane potential is a late adaptation of *Trypanosoma brucei brucei* to isometamidium preceded by mutations in the γ subunit of the F_1_F_0_-ATPase. PLoS Negl. Trop. Dis..

[102] Drew M.E., Morris J.C., Wang Z., Wells L., Sanchez M., Landfear S.M., Englund P.T. (2003). The adenosine analog tubercidin inhibits glycolysis in *Trypanosoma brucei* as revealed by an RNA interference library. J. Biol. Chem..

[103] Anyam J.V., Daikwo P.E., Ungogo M.A., Nweze N.E., Igoli N.P., Gray A.I., De Koning H.P., Igoli J.O. (2021). Two New Antiprotozoal Diterpenes from the Roots of *Acacia nilotica*. Front. Chem..

[104] Wallace L.J.M., Candlish D., De Koning H.P. (2002). Different substrate recognition motifs of human and trypanosome nucleobase transporters: Selective uptake of purine antimetabolites. J. Biol. Chem..

[105] Cheng Y., Prusoff W.H. (1973). Relationship between the inhibition constant (K_i_) and the concentration of inhibitor which causes 50 per cent inhibition (I_50_) of an enzymatic reaction. Biochem. Pharmacol..

