# Supplementary material for: Cloning and Characterization of Trypanosoma congolense and T. vivax Nucleoside Transporters Reveal the Potential of P1-Type Carriers for the Discovery of Broad-Spectrum Nucleoside-Based Therapeutics against Animal African Trypanosomiasis
Source: Int J Mol Sci. 2023 Feb 5;24(4):3144. doi: 10.3390/ijms24043144 (PMC9960827; doi:10.3390/ijms24043144)
Supplement: Supplementary file 1 [file ijms-24-03144-s001.zip › ijms-2184442-supplementary.pdf]

Supplemental Figure S1. Tree of nucleobase transporters from *T. congolense* IL-3000.

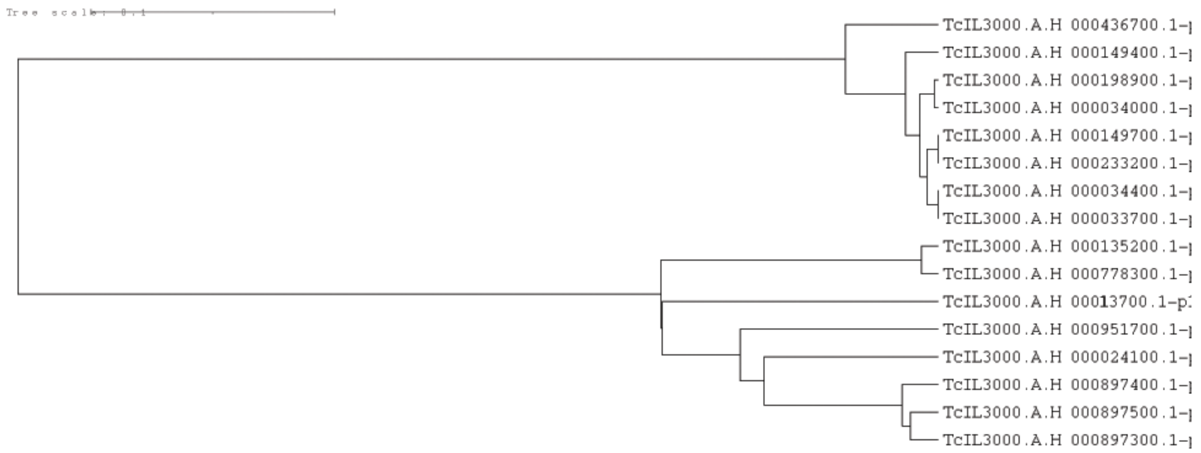

Tree was constructed by CLUSTAL Omega (1.2.3) multiple sequence alignment in TriTrypDB

Supplemental Figure S2. Multiple alignment of *T. congolense* nucleobase transporters IL300-2019 sequence from TriTrypDB
Made by CLUSTAL Omega (1.2.3) multiple sequence alignment in TriTrypDB

```
TcIL3000.A.H_000436700.1-p1      MASDTSFATKTYVCVTCFILDVCAYWAVETRLLAPIFMASHYKYITGNEHAEPSSVLFWL  60
TcIL3000.A.H_000149400.1-p1      MASDTSFATKTYVCVTCFILDVCAYWAVETRFLAPIFMASHYKYITGNEHAEPSSVLFWL  60
TcIL3000.A.H_000034000.1-p1      MASDTSFATKTYVCVTCFILDVCAYWAVETRFLAPIFMASHYKYITGNEHAEPSSVLFWL  60
TcIL3000.A.H_000198900.1-p1      MASDTSFATKTYVCVTCFILDVCAYWAVETRFLAPIFMASHYKYITGNEHAEPSSVLFWL  60
TcIL3000.A.H_000033700.1-p1      MASDTSFATKTYVCVTCFILDVCAYWAVETRFLAPIFMASHYKYITGNEHAEPSSVLFWL  60
TcIL3000.A.H_000034400.1-p1      MASDTSFATKTYVCVTCFILDVCAYWAVETRFLAPIFMASHYKYITGNEHAEPSSVLFWL  60
TcIL3000.A.H_000233200.1-p1      MASDTSFATKTYVCVTCFILDVCAYWAVETRFLAPIFMASHYKYITGNEHAEPSSVLFWL  60
TcIL3000.A.H_000149700.1-p1      MASDTSFATKTYVCVTCFILDVCAYWAVETRFLAPIFMASHYKYITGNEHAEPSSVLFWL  60
TcIL3000.A.H_000778300.1-p1      MALGFSSAAEVYVYVTCIFLGMSILLPLNTRYSAPMFMINYYKYVTGDENAEPNSKLFWA  60
TcIL3000.A.H_000135200.1-p1      MALGFSSAAEVYVYVTCIFLGMSILLPLNTRYSAPMFMINYYKYVTGDEDAEPNSKLFWA  60
TcIL3000.A.H_000113700.1-p1      -----------------------------------MFMMNYYKYVTGNEDAEPNSKLFWA  25
TcIL3000.A.H_000951700.1-p1      MALGLSSSAEVYVYVTCLFLGVSIILPLNTRYSAPMFMINYYKYVTGDENAKPNSVLFWA  60
TcIL3000.A.H_000024100.1-p1      MALGLSSSAEVYVYVTCIFLGVSILLPSSTRYSAPMFMVNYYKYVTGDENAEPNSVLFWA  60
TcIL3000.A.H_000897400.1-p1      MALGFSSSAEVYVYVTCLLLGMSILLPLNTRYSAPMFMVNYYKYVTGDENAEPNSVLFWA  60
TcIL3000.A.H_000897300.1-p1      MALGFSSSAEVYVYVTCLLLGVSILLPLNTRYSAPMFMVNYYKYVTGDENAEPNSVLFWA  60
TcIL3000.A.H_000897500.1-p1      MALGFSSSAEVYVYVTCLLLGVSILLPLNTRYSAPMFMVNYYKYVTGDENAEPNSVLFWA  60
                                                                    :** .:***:**:*.*:*.* ***

TcIL3000.A.H_000436700.1-p1      NIFTFHNLILFAARALAERPELERFVRGWGLRLRFTLSIVLILAELALIFFLPVLSMPLP  120
TcIL3000.A.H_000149400.1-p1      NIFTFHNLILFAARALAERPELERFVRGWGFRLRFTLSIVLILAELALIFFLPVLSMPLP  120
TcIL3000.A.H_000034000.1-p1      NIFTFYNLILFAARALAERPELERLVRGWGLRLRFTLSIVLILAELALIFFLPVLSMSPP  120
TcIL3000.A.H_000198900.1-p1      NIFTFYNLILFAARALAERPELERLVRGWGLRLRFTLSIVLILAELALIFFLPVLSMSPP  120
TcIL3000.A.H_000033700.1-p1      NIFTFHNLILFAARALAERPELERLVRGWGLRLRFTLSIVLILAELALIFFLPVLSMSPP  120
TcIL3000.A.H_000034400.1-p1      NIFTFHNLILFAARALAERPELERLVRGWGLRLRFTLSIVLILAELALIFFLPVLSMSPP  120
TcIL3000.A.H_000233200.1-p1      NIFTFHNLILFAARALAERPELERLVRGWGLRLRFTLSIVLILAELALIFFLPVLSMSPP  120
TcIL3000.A.H_000149700.1-p1      NIFTFHNLILFAARALAERPELERLVRGWGLRLRFTLSIVLILAELALIFFLPVLSMSPP  120
TcIL3000.A.H_000778300.1-p1      NILTFYGAISLVIQSLVGPTVLTQAARRLSLSVRFTLSITSMMIEVFVVLMMPVIKVSQT  120
TcIL3000.A.H_000135200.1-p1      NILTFYGAISLVIQSLVGPTVLTQAARRLSLSVRFTLSITSMMIEVFVVLMMPVIKVSQT  120
TcIL3000.A.H_000113700.1-p1      NILTFYGVISLVTQSLIGTAVFTSTICRVSLSVRFALSITFMMIEFFVVIMMPVIKVSQT  85
TcIL3000.A.H_000951700.1-p1      NILTFYTVISLVTQSFVGPTVLTQTVRRLSLSLRFALSITLMMTEVFMVLMLPITKVSQT  120
TcIL3000.A.H_000024100.1-p1      NILTFYTVISLVTQSFVGPTVLTQTVRRLSLSLRFTLSITLMMVEVFVVLMLPVIKVSQT  120
TcIL3000.A.H_000897400.1-p1      NILTFYNVISLVTQSLIGPTVLTPTVRRLSLSLRFALSITFMMMEVFVVLMLPVIKVSQT  120
TcIL3000.A.H_000897300.1-p1      NILTFYNVISLVTQSLIGPTVLTPTVRRLSLSLRFALSITFMMMEVFVILMLPVIKVSQT  120
TcIL3000.A.H_000897500.1-p1      NILTFYNVISLVTQSLIGPTVLTPTVRRLSLSLRFALSITFMMMEVFVILMLPVIKVSQT  120
                                 **:**:  * :. :::     :       .: :**:***. :: *. :::::*: .:

TcIL3000.A.H_000436700.1-p1      NAIVSLCFTTILGTIGKQFLGRTTHTLWDFLLTEKAGGVIRLTAFLVAIVHATLCIIVVV  180
TcIL3000.A.H_000149400.1-p1      NAIVSLCFTTILGTIGKQFLGRTTHTLWDFLLTEKAGGVIRLTAFLVAIVHAMLCIIVVV  180
TcIL3000.A.H_000034000.1-p1      YAIVSLCFTTILGTIGKQFLGRTTHTLWDFLLTEKAGGVMRFTAFLVAIVHATLCIIVVV  180
TcIL3000.A.H_000198900.1-p1      YAIVSLCFTTILGTIGKQFLGRTTHTLWDFLLTEKAGGVMRFTAFLVAIVHATLCIIVVV  180
TcIL3000.A.H_000033700.1-p1      YAIVSLCFTTILGTIGKQFLGRTTHTLWDFLLTEKAGGVIRLTAFLVAIVHAMLCIIVVV  180
TcIL3000.A.H_000034400.1-p1      YAIVSLCFTTILGTIGKQFLGRTTHTLWDFLLTEKAGGVIRLTAFLVAIVHAMLCIIVVV  180
TcIL3000.A.H_000233200.1-p1      YAIVSLCFTTILGTIGKQFLGRTTHTLWDFLLTEKAGGVIRLTAFLVAIVHATLCIIVVV  180
TcIL3000.A.H_000149700.1-p1      YAIVSLCFTTILGTIGKQFLGRTTHTLWDFLLTEKAGGVIRLTAFLVAIVHATLCIIVVV  180
TcIL3000.A.H_000778300.1-p1      TAIVFFVIVTIVGGIGKCYLEATCYTLVAPLPPKFMSTIMFGICFCGVVTSTIQCIIKAS  180
TcIL3000.A.H_000135200.1-p1      TAIVFFVIVTIVGGIGKCYLEATCYTLVAPLPPKFMSTIMFGICFCGVVTSTIQCIIKAS  180
TcIL3000.A.H_000113700.1-p1      TAIVFFVIVTIVCGIWKCYLEATCYTLVAHVPPKFMSTIMFSICFCGAITSTIRCIIKAS  145
TcIL3000.A.H_000951700.1-p1      TAIVFFVIATILGGMGKPCLEATSYTLAASMPEKFTSTIMFGISICGLLTSMMQCIVKLS  180
TcIL3000.A.H_000024100.1-p1      VAIVFFVIVTIVGGIGKCYLEATCYTLVAPLPPKFMSTIMFGISICGVLTSTMQCIIKVS  180
TcIL3000.A.H_000897400.1-p1      VAIVFFVIVTILGGMGKSYLEATSYTLAASMPSKFMSTIMFGISICGVLTSTMQCIIKVS  180
TcIL3000.A.H_000897300.1-p1      VAIVFFVIVTILGGMGKCYLEATSYTLAASMPSKFMSTIMFGISFCGMLTSTMQCIIKVS  180
TcIL3000.A.H_000897500.1-p1      VAIVFFVIVAILGGMGKCYLEATSYTLAASMPSKFMSTIMFGISICGVLTSTMQCIIKVS  180
                                  *** : :.:*:  : *  *  * :**   :  :  . ::   .:   :.    **:

TcIL3000.A.H_000436700.1-p1      PGTTGESRENTLSLHTVVYAVVLVIALVMVLSLSLIRCTREGFRSAQRAAQKGRVEGINH  240
TcIL3000.A.H_000149400.1-p1      PGTTGESRENTLSLHTFVYAVVLVIALVMVLSLSLIRCTREGFRSAQRAAQKGRVEGINH  240
TcIL3000.A.H_000034000.1-p1      PGTTGESRENTLSLHTVVYAVVLVIALVMVLSLSLIRCTREGFRSAQRAAQKGRVEGINH  240
TcIL3000.A.H_000198900.1-p1      PGTTGESRENTLSLHTVVYAVVLLIALVMVLSLSLIRCTREGFRSAQRAAQKGRVEGINH  240
TcIL3000.A.H_000033700.1-p1      PGTTGESRENTLSLHTVVYAVVLVIALVMVLSLALIRCTREGFRSAQRAAQKGRVEGINH  240
TcIL3000.A.H_000034400.1-p1      PGTTGESRENTLSLHTVVYAVVLVIALVMVLSLALIRCTREGFRSAQRAAQKGRVEGINH  240
TcIL3000.A.H_000233200.1-p1      PGTTGESRENTLSLHTVVYAVVLVIALVMALSLSLIRCTREGFRSAQRAAQKGRVEGINH  240
TcIL3000.A.H_000149700.1-p1      PGTTGESRENTLSLHTVVYAVVLVIALVMALSLSLIRCTREGFRSAQRAAQKGRVEGINH  240
TcIL3000.A.H_000778300.1-p1      MEDTYESHLTQSYIYFSLALLFMAAALVMALSLRYNSYAQEMVAEYRVLKQKQEGQKVEP  240
TcIL3000.A.H_000135200.1-p1      MEDTYESHLTQSYIYFSLALLFMAAALVMALSLRYNSYAQEMVAEYRVLKQKQEGQKVEP  240
TcIL3000.A.H_000113700.1-p1      MEDTYESHLTQSYIYFSLALLIMAVALVMVLTLHYNSYAQQHVAEFRVLKQKHDENNVEP  205
TcIL3000.A.H_000951700.1-p1      MENIYESQLTQSYIYFSLALLIMAVALVMALSLRYNSYAQEHVAEFRALKQKHDDNKVEP  240
TcIL3000.A.H_000024100.1-p1      MENTYDSQLTQSYIYFSLSLLIMAVALVMVLSLRYNSYAQEYVAEFRVLTQKQEDEGVEP  240
TcIL3000.A.H_000897400.1-p1      MENTYESQLTQSYIYFSLALLIMAVALVMVLTLRYNSYAQEHVAEFRVLKQKQEDEGVEP  240
TcIL3000.A.H_000897300.1-p1      MENTYESQLTQSYIYFSLALLIMAVALVMVLTLRYNSYAQEHVAEFRVLKQKQEDEGVEP  240
TcIL3000.A.H_000897500.1-p1      MENTYESQLTQSYIYFSLALLIMAVALVMVLTLRYNSYAQEHVAEFRVLKQKQEDEGVEP  240
                                      :*: .   ::  :  :.:  ****.*:*     ::: . . :   **   : ::

TcIL3000.A.H_000436700.1-p1      LIERVGKTR-KTLESIGVRLQAQRNPIRRKEMKKKHRQAGEQTLEAQEDIRRR-------  292
TcIL3000.A.H_000149400.1-p1      LIERVGKTR-KTLESIRVRLHAQSDPILRNEVQKEHQQAKRETVEAQEDIRRR-------  292
```

```
TcIL3000.A.H_000034000.1-p1      LIERVGKTR-KTLESIRVRLHAQSDPILRNEVQKEHQQAKRETVEAQEDIRRR------- 292
TcIL3000.A.H_000198900.1-p1      LIERVGKTR-KTLESIRVRLHAQSDPILRNEVQKEHQQAKRETVEAQEDIRRR------- 292
TcIL3000.A.H_000033700.1-p1      LIERVGKTR-KTLESIRVRLHAQSDPILRNEVQKEHQQAKRETVEAQEDIRRR------- 292
TcIL3000.A.H_000034400.1-p1      LIERVGKTR-KTLESIRVRLHAQSDPILRNEVQKEHQQAKRETVEAQEDIRRR------- 292
TcIL3000.A.H_000233200.1-p1      LIERVGKTR-KTLESIRVRLHAQSDPILRNEVQKEHQQAKRETVEAQEDIRRR------- 292
TcIL3000.A.H_000149700.1-p1      LIERVGKTR-KTLESIRVRLHAQSDPILRNEVQKEHQQAKRETVEAQEDIRRR------- 292
TcIL3000.A.H_000778300.1-p1      Q--PVADVPKEVKEPTDGEED----PQ----NKEENKGEGEVGMTTAEQLAATAVMPVVK 290
TcIL3000.A.H_000135200.1-p1      Q--PVADVPTEGKEPTCGEEG----PQ----NKEESKGEGEVGMTTAEQLAATAVMPVVK 290
TcIL3000.A.H_000113700.1-p1      Q--PVADVPKEVKEPTGCEDK----C--------DVDSKGEGCMTTAEQLAATPIMPMVR 251
TcIL3000.A.H_000951700.1-p1      Q--TAADVPTEMKETTDVEDK----S--------DVDSRGEGGMTTAEQLVATPIMPVVR 286
TcIL3000.A.H_000024100.1-p1      Q--PVADVPTEVKESTSCEDK----C--------DVDSKEEGCITTAEQLSPTPIMPVVR 286
TcIL3000.A.H_000897400.1-p1      Q--PVADVPTEVKEPTGVEDQ----C--------EVDNKGEGGMTTAEQLAATPIMPVVR 286
TcIL3000.A.H_000897300.1-p1      Q--PVADVPTEVKEPTGVEDQ----C--------EVDNSGEGGMTTAEQLAATPIMPVVR 286
TcIL3000.A.H_000897500.1-p1      Q--PVADVPTEVKEPTGVEDQ----C--------EVDNKGEGGMTTAEQLAATPIMPVVR 286
                                     ....  :  *    .               .     .  : : *::

TcIL3000.A.H_000436700.1-p1      LKDEASISAL-------------SRLAEFEAEWARPLPPEASSDGGNRDVAYSPQVKRAD 339
TcIL3000.A.H_000149400.1-p1      LKDEASISAL-------------SRLAEFEAEWARPLPPEASSDGGNRDVAQSPQAPDVV 339
TcIL3000.A.H_000034000.1-p1      LKDEASISAL-------------SRLAEFEAEWARPLPPEASSDGGNRDVAQSPQAPDVV 339
TcIL3000.A.H_000198900.1-p1      LKDEASISAL-------------SRLAEFEAEWARPLPPEASSDGGNRDVAQSPQAPDVV 339
TcIL3000.A.H_000033700.1-p1      LKDEASISAL-------------SRLAEFEAEWARPLPPEASSDGGNRDVAQSPQAPDVV 339
TcIL3000.A.H_000034400.1-p1      LKDEASISAL-------------SRLAEFEAEWARPLPPEASSDGGNRDVAQSPQAPDVV 339
TcIL3000.A.H_000233200.1-p1      LKDEASISAL-------------SRLAEFEAEWARPLPPEASSDGGNRDVAQSPQAPDVV 339
TcIL3000.A.H_000149700.1-p1      LKDEASISAL-------------SRLAEFEAEWARPLPPEASSDGGNRDVAQSPQAPDVV 339
TcIL3000.A.H_000778300.1-p1      MIHMMLLSCFLGFFVTIFIFPNLIIPIDRTHKWFATIAALIFNIG-KSIGSFSTS----- 344
TcIL3000.A.H_000135200.1-p1      MIHMMLLSCFLGFFVTIFIFPNLIIPIDRTHKWFATIAALIFNIG-KSIGSFSTS----- 344
TcIL3000.A.H_000113700.1-p1      IIRTVLISCFLGFFLTIFIFPTLVVPIDRVDDWFSTIAALVYNIG-YSIGSFSTS----- 305
TcIL3000.A.H_000951700.1-p1      IIRMMLISCFLGFFLTIFIFPSLIIPIDRADDWFSTIAALIFNIG-NSIGSFSTS----- 340
TcIL3000.A.H_000024100.1-p1      IIRVMLISCFLGFFLTIFIFPSFGIPIDRVDDWFSTIAALIFNVG-KSIGSFSTS----- 340
TcIL3000.A.H_000897400.1-p1      IIRMMLISCFLGFFLTIFIFPSLVIPIDRVDDWFSTIAALVYNIG-NSIGSFSTS----- 340
TcIL3000.A.H_000897300.1-p1      IIRMMLISCFLGFFLTIFIFPSLVIPIDRVDDWFSTIAALIFNIG-NSIGAFSTS----- 340
TcIL3000.A.H_000897500.1-p1      IIRMMLISCFLGFFLTIFIFPSLVIPIDRVDDWFSTIAALIFNIG-NSIGAFSTS----- 340
                                 :     :*.:                 :   .*   :     . *     : * .

TcIL3000.A.H_000436700.1-p1      LRQKSTPRPLASS----------------------------------------------- 352
TcIL3000.A.H_000149400.1-p1      ------------------------------------------------------------ 339
TcIL3000.A.H_000034000.1-p1      ------------------------------------------------------------ 339
TcIL3000.A.H_000198900.1-p1      ------------------------------------------------------------ 339
TcIL3000.A.H_000033700.1-p1      ------------------------------------------------------------ 339
TcIL3000.A.H_000034400.1-p1      ------------------------------------------------------------ 339
TcIL3000.A.H_000233200.1-p1      ------------------------------------------------------------ 339
TcIL3000.A.H_000149700.1-p1      ------------------------------------------------------------ 339
TcIL3000.A.H_000778300.1-p1      FKMFMYPRGVVLYGSIVRFLLIIPFMLSIYKHIPGHTVPYIFSFILGLTHYFPVLSMVYG 404
TcIL3000.A.H_000135200.1-p1      FKMFMYPRGVVLYGSIVRFLLIIPFMLSIYKHIPGHTVPYIFSFILGLTHYFPVLSMVYG 404
TcIL3000.A.H_000113700.1-p1      FETFRYPRRVVLYGS--------------------------------------------- 320
TcIL3000.A.H_000951700.1-p1      FETFRYPRRVVLYVSIVRFLLIIPFILSIYKHIPGHTVPYIFSFILGLTHYFPVLSMVYG 400
TcIL3000.A.H_000024100.1-p1      SEMFMHPRGVALYWSIVRFLLIIPFMLSIYKHITGHVVPYIFSFILGLTHQVSVLSMVYG 400
TcIL3000.A.H_000897400.1-p1      FETFRYPRRVVLYGSIVRFLLIIPFVLSIYKYIPGHVGPYIFSFILGLTHQVAVLSMVYG 400
TcIL3000.A.H_000897300.1-p1      FETFRYPRRVVLYGIIVRFLLIIPFMLSIYKYIPGHVGPYIFSFILGLTHQVPVLSMVYG 400
TcIL3000.A.H_000897500.1-p1      FETFRYPRRVVLYGSIVRFLLIIPFVLSIYKYIPGHVGPYIFSFILGLTHQVAVLSMVYG 400


TcIL3000.A.H_000436700.1-p1      ------------------------------------------- 352
TcIL3000.A.H_000149400.1-p1      ------------------------------------------- 339
TcIL3000.A.H_000034000.1-p1      ------------------------------------------- 339
TcIL3000.A.H_000198900.1-p1      ------------------------------------------- 339
TcIL3000.A.H_000033700.1-p1      ------------------------------------------- 339
TcIL3000.A.H_000034400.1-p1      ------------------------------------------- 339
TcIL3000.A.H_000233200.1-p1      ------------------------------------------- 339
TcIL3000.A.H_000149700.1-p1      ------------------------------------------- 339
TcIL3000.A.H_000778300.1-p1      PTTPGLDDGQKLMAGQLMGIALLVGVSAASVVAMIVVIFLPQD 447
TcIL3000.A.H_000135200.1-p1      PTTPGLDDGQKLMAGQLMGIALLVGVSAASVVAMIVVIFLPQD 447
TcIL3000.A.H_000113700.1-p1      ------------------------------------------- 320
TcIL3000.A.H_000951700.1-p1      PVTHGLNDGQKLMAGRLMGMALLLGASAASVVAMTVVIFLP-- 441
TcIL3000.A.H_000024100.1-p1      PITTGLNDGQKLIEGQLIGIALLVGASAASVAAMVVVIFLPRD 443
TcIL3000.A.H_000897400.1-p1      PITPGLNDGQKLMTGQLMGIALLVGASAASVVAMIVVIFLP-- 441
TcIL3000.A.H_000897300.1-p1      PITPGLNDGQKLMAGQLMGIALLAGATAASVSAMIVVIFLP-- 441
TcIL3000.A.H_000897500.1-p1      PITPGLNDGQKLMAGQLMSIALLAGASAASVVAMIVVIFLP-- 441
```

Supplemental Table S1. *T. congolense* nucleobase gene family

IL3000 (2019) sequences and IDs from TriTrypDB (https://tritrypdb.org/tritrypdb/app)

TMD prediction: https://services.healthtech.dtu.dk/service.php?TMHMM-2.0
E value for ENT family from Panther via TriTrypDB

TriTrypDB gene ID	a.a.	TMDs	E-value for ENT
TcIL3000.A.H_000436700.1-p1	352	6	1.7E-19
TcIL3000.A.H_000149400.1-p1	339	6	2.0E-19
TcIL3000.A.H_000034000.1-p1	339	6	1.6E-21
TcIL3000.A.H_000198900.1-p1	339	6	1.6E-21
TcIL3000.A.H_000033700.1-p1	339	6	2.0E-19
TcIL3000.A.H_000034400.1-p1	339	6	2.0E-19
TcIL3000.A.H_000233200.1-p1	339	6	2.0E-19
TcIL3000.A.H_000149700.1-p1	339	6	2.3E-19
TcIL3000.A.H_000778300.1-p1	447	11	0
TcIL3000.A.H_000135200.1-p1	447	11	0
TcIL3000.A.H_000113700.1-p1	320	7	4.5E-95
TcIL3000.A.H_000951700.1-p1	441	10	0
TcIL3000.A.H_000024100.1-p1	443	10	0
TcIL3000.A.H_000897400.1-p1	441	11	0
TcIL3000.A.H_000897300.1-p1	441	11	0
TcIL3000.A.H_000897500.1-p1	441	11	0

Supplementary Table S2: List of primers used, their sequences and purposes

Primer	Sequence	Function	Restriction site
HDK1761	CTTCCACTTGTCAAGCGAATTC**CATATG**ATGCTCGGTTTTGAATCCATTTCCG	Forward primer for TcoAT1	*Nde*I
HDK1762	CTCAGTGGTGGTGGTGGTGGTG**CTCGAG**CCACTCTGGCAGGGCC	Reverse primer for TcoAT1	*Xho*I
HDK1763	CTTCCACTTGTCAAGCGAATTC**CATATG**ATGGCAGTGTTAGGGTTTGAGTCC	Forward primer for TvY486_0202110	*Nde*I
HDK1764	CTCAGTGGTGGTGGTGGTGGTG**CTCGAG**CAGCGTCTTGCTCAGTCCTATTGC	Reverse primer for TvY486_0202110	*Xho*I
HDK1765	CTTCCACTTGTCAAGCGAATTC**CATATG**ATGCTAGGCTTCGAGTCCACCTC	Forward primer for TvY486_1112030	*Nde*I
HDK1766	CTCAGTGGTGGTGGTGGTGGTG**CTCGAG**AAGCAACTTGCTCAGTCCTATTGC	Reverse primer for TvY486_1112030	*Xho*I
HDK1767	CTTCCACTTGTCAAGCGAATTC**CATATG**ATGGGGTTACTGGGGTTTGAGTCG	Forward primer for TvY486_0043680	*Nde*I
HDK1768	CTCAGTGGTGGTGGTGGTGGTG**CTCGAG**CAAGTGCTTCTCCAAACCCAGTGC	Reverse primer for TvY486_0043680	*Xho*I
HDK1769	CTTCCACTTGTCAAGCGAATTC**CATATG**ATGGGGTTGCTGGGTTTTGAGTCG	Forward primer for TvY486_0014570	*Nde*I
HDK1770	CTCAGTGGTGGTGGTGGTGGTG**CTCGAG**CCGTCCGCGGGATGGGTG	Reverse primer for TvY486_0014570	*Xho*I
HDK1734	CGACCTTACACCTCTACTTGTC	Reverse primer for pNUS-HcN plasmid	
HDK340	CGTGGAGCAGCTGAAGGACA	Reverse primer for pNUS-HcN plasmid	

Sequence in bold represent restriction sites. Sequence in lower case represent intended mutation.
